# Combined and progestagen-only hormonal contraceptives and breast cancer risk: A UK nested case–control study and meta-analysis

**DOI:** 10.1371/journal.pmed.1004188

**Published:** 2023-03-21

**Authors:** Danielle Fitzpatrick, Kirstin Pirie, Gillian Reeves, Jane Green, Valerie Beral

**Affiliations:** 1 Cancer Epidemiology Unit, Nuffield Department of Population Health, University of Oxford, Oxford, United Kingdom; 2 Adelaide Medical School, Faculty of Health and Medical Sciences, University of Adelaide, South Australia, Australia

## Abstract

**Background:**

Current or recent use of combined oral contraceptives (containing oestrogen+progestagen) has been associated with a small increase in breast cancer risk. Progestagen-only contraceptive use is increasing, but information on associated risks is limited. We aimed to assess breast cancer risk associated with current or recent use of different types of hormonal contraceptives in premenopausal women, with particular emphasis on progestagen-only preparations.

**Methods and findings:**

Hormonal contraceptive prescriptions recorded prospectively in a UK primary care database (Clinical Practice Research Datalink [CPRD]) were compared in a nested case–control study for 9,498 women aged <50 years with incident invasive breast cancer diagnosed in 1996 to 2017, and for 18,171 closely matched controls. On average, 7.3 (standard deviation [SD] 4.6) years of clinical records were available for each case and their matched controls prior to the date of diagnosis. Conditional logistic regression yielded odds ratios (ORs) and 95% confidence intervals (CIs) of breast cancer by the hormonal contraceptive type last prescribed, controlled for age, GP practice, body mass index, number of recorded births, time since last birth, and alcohol intake. MEDLINE and Embase were searched for observational studies published between 01 January 1995 and 01 November 2022 that reported on the association between current or recent progestagen-only contraceptive use and breast cancer risk in premenopausal women. Fixed effects meta-analyses combined the CPRD results with previously published results from 12 observational studies for progestagen-only preparations.

Overall, 44% (4,195/9,498) of women with breast cancer and 39% (7,092/18,171) of matched controls had a hormonal contraceptive prescription an average of 3.1 (SD 3.7) years before breast cancer diagnosis (or equivalent date for controls). About half the prescriptions were for progestagen-only preparations. Breast cancer ORs were similarly and significantly raised if the last hormonal contraceptive prescription was for oral combined, oral progestagen-only, injected progestagen, or progestagen-releasing intrauterine devices (IUDs): ORs = 1.23 (95% CI [1.14 to 1.32]; *p* < 0.001), 1.26 (95% CI [1.16 to 1.37]; *p* < 0.001), 1.25 (95% CI [1.07 to 1.45]; *p* = 0.004), and 1.32 (95% CI [1.17 to 1.49]; *p* < 0.001), respectively. Our meta-analyses yielded significantly raised relative risks (RRs) for current or recent use of progestagen-only contraceptives: oral = 1.29 (95% CI [1.21 to 1.37]; heterogeneity χ^2^_5_ = 6.7; *p* = 0.2), injected = 1.18 (95% CI [1.07 to 1.30]; heterogeneity χ^2^_8_ = 22.5; *p* = 0.004), implanted = 1.28 (95% CI [1.08 to 1.51]; heterogeneity χ^2^_3_ = 7.3; *p* = 0.06), and IUDs = 1.21 (95% CI [1.14 to 1.28]; heterogeneity χ^2^_4_ = 7.9; *p* = 0.1). When the CPRD results were combined with those from previous published findings (which included women from a wider age range), the resulting 15-year absolute excess risk associated with 5 years use of oral combined or progestagen-only contraceptives in high-income countries was estimated at: 8 per 100,000 users from age 16 to 20 years and 265 per 100,000 users from age 35 to 39 years. The main limitation of the study design was that, due to the nature of the CPRD data and most other prescription databases, information on contraceptive use was recorded during a defined period only, with information before entry into the database generally being unavailable. This means that although our findings provide evidence about the short-term associations between hormonal contraceptives and breast cancer risk, they do not provide information regarding longer-term associations, or the impact of total duration of contraceptive use on breast cancer risk.

**Conclusions:**

This study provides important new evidence that current or recent use of progestagen-only contraceptives is associated with a slight increase in breast cancer risk, which does not appear to vary by mode of delivery, and is similar in magnitude to that associated with combined hormonal contraceptives. Given that the underlying risk of breast cancer increases with advancing age, the absolute excess risk associated with use of either type of oral contraceptive is estimated to be smaller in women who use it at younger rather than at older ages. Such risks need be balanced against the benefits of using contraceptives during the childbearing years.

## Abstract 2

In a UK nested case-control study and subsequent meta-analysis, Kirstin Pirie and colleagues explore the association between combined and progestogen-only hormonal contraceptives and the risk of breast cancer.

## Author summary

**Why was this study done?:**

- Use of combined oral contraceptives has been associated with a small transient increase in breast cancer risk, but there is limited data about the effect of progestagen-only contraceptives on breast cancer risk.
- Use of progestagen-only hormonal contraceptives has increased substantially over the last decade, and in 2020, there were almost as many prescriptions in England for oral progestagen-only contraceptives as for combined oral contraceptives.
- Given the increasing use of progestagen-only contraceptives, it is important to understand how their use is associated with breast cancer risk.

**What did the researchers do and find?:**

- We carried out a nested case–control study in the Clinical Practice Research Datalink (CPRD), including almost 10,000 women aged <50 years with breast cancer, to assess the relationship between a woman’s recent use of hormonal contraceptives and her subsequent risk of breast cancer.
- In our study, current or recent use of hormonal contraceptives was associated with a similarly increased risk of breast cancer regardless of whether the preparation last used was oral combined, oral progestagen-only, injectable progestagen, progestagen implant, or progestagen intrauterine device.
- When our findings for progestagen-only contraceptives were combined with those of previous studies, there was evidence of a broadly similar increased risk of breast cancer in current and recent users of all four types of progestagen-only preparations.

**What do these findings mean?:**

- Our findings suggest that there is a relative increase of around 20% to 30% in breast cancer risk associated with current or recent use of either combined oral or progestagen-only contraceptives.
- When our findings for oral contraceptives are combined with results from previous studies (which included women in a wider age range), they suggest that the 15-year absolute excess risk of breast cancer associated with use of oral contraceptives ranges from 8 per 100,000 users (an increase in incidence from 0.084% to 0.093%) for use from age 16 to 20 to about 265 per 100,000 users (from 2.0% to 2.2%) for use from age 35 to 39.
- These excess risks must be viewed in the context of the well-established benefits of contraceptive use in women’s reproductive years.
- The lack of complete information on a woman’s prescription history means that this study was unable to assess the long-term associations of contraceptive use on breast cancer risk, but this should not have unduly affected the findings regarding their short-term associations.

## Introduction

A meta-analysis of the worldwide evidence on breast cancer risk associated with use of combined (containing oestrogens plus progestagens) oral contraceptives in 1996 found a slightly increased risk in current or recent users that declined after use ceased, with no apparent excess risk 10 or more years after cessation [1]. At that time, there was limited information on risks associated with hormonal contraceptives containing only progestagens. Published evidence since then on premenopausal breast cancer risk associated with use of progestagen-only contraceptives is limited [2–12].

Use of the different types of hormonal contraceptives has changed over time, with recent increases in use of progestagen-only preparations, both as oral and as long-acting parenteral formulations such as injectables, implants, and progestagen-releasing intrauterine devices (IUDs). In England, for example, prescriptions for oral progestagen-only contraceptives almost doubled in the last decade (from 1.9 to 3.3 million from 2010 to 2020); and in 2020, there were almost as many prescriptions for oral progestagen-only contraceptives as for oral combined contraceptives (3.3 million of each) [13]. Given the trend towards increasing use of progestagen-only contraceptives, it is important to reliably quantify their effects on breast cancer risk.

We aimed to assess breast cancer risk associated with current or recent use of different types of hormonal contraceptives in premenopausal women, with particular emphasis on progestagen-only preparations. We present new data on breast cancer risk associated with prospectively recorded prescriptions for hormonal contraceptives in women aged <50 years in the United Kingdom (UK) primary care Clinical Practice Research Datalink (CPRD) and conduct meta-analyses of breast cancer risk associated with current or recent progestagen-only hormonal contraceptives, combining the new and previously published findings.

## Methods

This study is reported as per the Strengthening the Reporting of Observational Studies in Epidemiology (STROBE) guideline (S1 STROBE Checklist) and the Preferred Reporting Items for Systematic Reviews and Meta-Analyses (PRISMA) guideline (S1 PRISMA Checklist). All analyses were done in STATA version 17.0, and graphs were generated using the R package Jasper [14].

### Clinical Practice Research Datalink (CPRD)

The CPRD is a computerised UK primary care database containing anonymised, linked, and prospective medical records for approximately 11 million individuals registered with a National Health Service (NHS) general practitioner (GP) [15]. As of 2013, approximately 7% of the UK population were active participants in CPRD [15]. CPRD’s Independent Scientific Advisory Committee approved the study protocol in 2011 (10_152), with an amendment for an updated dataset approved in 2017 (S1 Protocol).

### Study design

The association between use of hormonal contraceptives and invasive breast cancer risk in CPRD was studied using a nested case–control design. Although the original protocol allowed for the assessment of hormonal contraceptive use in relation to both in situ and invasive breast cancer, we present findings here for invasive breast cancer only since this was the primary outcome of interest. Cases are all women aged 20 to 49 years with incident invasive breast cancer recorded between 1 January 1996 and 20 September 2017, with no prior record of incident in situ breast cancer. Invasive breast cancer was defined using CPRD Read codes for the disease (S1 File) [16]. Oestrogen-receptor (ER) status of the tumours was not recorded, and so this was assessed by the presence of one of more prescriptions for tamoxifen and/or aromatase inhibitors up to 3 years after the cancer diagnosis date; for those with <12 months follow-up after diagnosis date, ER status was classified as unknown.

For each case, the “observation period” (the period during which reliable prescription data were available before diagnosis) was defined as starting either from 1 January 1995 or from the date of entry into an up-to-standard CPRD practice (whichever was later) and ending at the date of diagnosis. Two controls were selected for each case, matched on index date (date of diagnosis of the case), year of birth (+/−2 years), general practice, and observation period (the duration of observation prior to the index date for the control had to be at least as long as that of the case). Controls were selected from women with no record of invasive or in situ breast cancer before 20 September 2017. The resulting sample size was deemed sufficient to detect relevant effect sizes (S1 Protocol). To ensure identical opportunities for ascertainment of prescribing in cases and controls, the observation period for each matched control was truncated to be exactly the same time period as for the matched case. Both cases and controls were required to have a minimum of 12 months of follow-up prior to the index date.

Women were defined as having a prescription for hormonal contraceptives if they had one or more prescriptions for any hormonal contraceptive during the observation period. Nonusers were defined as women having no such prescription. We used the British National Formulary system (BNF sections 7.3.1 and 7.3.2 [17]) to classify the hormonal contraceptive preparation last prescribed: oral combined contraceptive, oral progestagen-only contraceptive, injectable progestagen, progestagen implant, or progestagen-releasing IUD. Current users of oral contraceptives were defined as women whose last prescription was <12 months prior to the index date; their duration of use during the observation window was calculated as the time between the first and last recorded prescription. Prescriptions for nonhormonal copper IUDs (BNF section 7.3.4 [17]) were also extracted. The small number of women whose last prescription was the combined contraceptive vaginal ring or the combined contraceptive patch were classified as other users. Prescriptions for emergency contraceptives were not included. Cases and controls with one or more prescriptions for hormone therapy for the menopause (BNF section 6.4.1.1 [17]; 2,032 women in total) were excluded since such women are likely to be postmenopausal, which would confound comparisons.

### Statistical analysis

A matched analysis was done using conditional logistic regression to calculate odds ratios (ORs) and 95% confidence intervals (CIs) for incident invasive breast cancer in women with one or more hormonal contraceptive prescriptions compared to women with no such prescription during the observation period. We also examined ORs separately in women with one or more hormonal contraceptive prescriptions by type of preparation last prescribed. Previous evidence suggests that the effect of hormonal contraceptive use on breast cancer risk lasts for up to 10 years after use ceases [1]. In order to assess the impact of potential confounding by prior use of other types of hormonal contraceptives, therefore, we further examined risks in the subset of women with an observation period of at least 10 years and no recorded use of other hormonal contraceptives within the observation window. All analyses were adjusted for number of recorded births (0, 1 to 2, 3+ births recorded before the index date, which included births before the observation period), time since last recorded birth (<5, 5 to 10 years, no record of birth within the observation period), body mass index (BMI <20, 20 to 24.9, 25 to 29.9, 30+ kg/m^2^), and alcohol intake (non/past drinker, drinker). For alcohol intake and BMI, we used the most recent record in the 10 years prior to 6 months before the index date. All of these adjustment variables had been specified in the original study protocol except for time since last birth, which was additionally adjusted for due to its observed association with case control status in these data, and its likely relationship with recent use of hormonal contraceptives. In a slight deviation from the original protocol, no adjustment was made for smoking status as it was not considered to be a substantial risk factor for breast cancer in premenopausal women. Women with missing values were assigned to a separate category, and sensitivity analyses were done restricting analyses to women with known values for these variables. Likelihood ratio tests were used to assess evidence of heterogeneity in risks across subgroups of women.

Other sensitivity analyses assessed robustness of results with respect to use of any hormonal contraceptive: by restricting the definition of hormonal contraceptive exposure to at least 2 prescriptions for hormonal contraceptives; by restricting analyses to women with an observation period of ≥5 years; and by excluding women with a history of hysterectomy, tubal ligation, and/or bilateral oophorectomy. To assess possible bias associated with those prescribed hormonal contraceptives having more frequent contact with GPs than the average, we also examined the relationship between breast cancer risk and other regularly prescribed medications that were not expected to be associated with breast cancer risk: nonsedating antihistamines, antibacterial eye preparations, and corticosteroids for asthma and other respiratory conditions.

The absolute excess incidence of breast cancer in women who used any type of oral contraceptive at different ages was estimated by applying relative risks (RRs) by time since last oral contraceptive use (combining the CPRD results with previously published findings; [1]) to breast cancer incidence rates in nonusers of hormonal contraceptives, using UK age-specific breast cancer rates, which are typical for rates in high-income countries (S2 File) [18].

### Meta-analysis

In November 2022, two authors (DF and KP) independently searched MEDLINE/PubMed and Embase for studies published between 1 January 1995 and 1 November 2022 that reported RRs and CIs for breast cancer associated with current and/or recent use of progestagen-only contraceptives compared to never-use in premenopausal women. Information was extracted independently by both DF and KP, and reference lists of included studies and relevant systematic reviews were searched for further references. The following search terms were used:

MEDLINE//Pubmed:

(("contraceptive agents"[MeSH Terms] OR "contraceptive devices"[MeSH Terms] OR "contracept*"[Title/Abstract] OR "intrauterine device*"[Title/Abstract] OR "IUD"[Title/Abstract]) AND ("breast neoplasms"[MeSH Terms] OR ("breast"[Title/Abstract] AND ("tumour*"[Title/Abstract] OR "tumor*"[Title/Abstract] OR "cancer*"[Title/Abstract] OR "carcinoma*"[Title/Abstract] OR "malignan*"[Title/Abstract]))) AND 1995/01/01:2022/11/01[Date—Publication]) NOT ("case reports"[Publication Type] OR "editorial"[Publication Type] OR "letter"[Publication Type] OR "comment"[Publication Type])

Embase:

1. intrauterine contraceptive device/
2. contraception/ or contraceptive agent/ or contracept*.mp.
3. IUD.mp.
4. 1 or 2 or 3
5. breast cancer/
6. 4 and 5
7. limit 6 to human
8. limit 7 to yr = "1995 -Current"
9. limit 8 to adult <18 to 64 years>

Eligible studies are those that reported RRs and CIs for breast cancer associated with current or recent use of progestagen-only contraceptives in premenopausal women. Studies that lacked information on recency of use, were restricted to specific patient populations, or included postmenopausal women only were excluded. Information was extracted from each of the included study reports by one reviewer (DF) and checked by another (KP). Information was sought on study characteristics (country, year of publication, study design), study participants (age at diagnosis, menopausal status), analysis characteristics (exposure definition, adjustment factors used), and study results (number of exposed/unexposed cases, RRs and CIs). This information was tabulated in order to confirm that each study met the eligibility criteria, and to enable assessment of each study in terms of factors that may lead to biased estimates, such as study design and adjustment for confounding. Sensitivity analyses explored the likely impact of potential sources of bias by restricting analyses to studies with particular characteristics. Funnel plots were produced to assess small study effects. This literature review was not registered, and a protocol was not prepared. Where necessary, results reported by fine categories of time since last use were combined in order to produce categories that were more comparable with other studies [19]. Summary RRs, combining study-specific results, were calculated as weighted averages with weights proportional to the inverse of the variance of the study-specific log RR. Chi-squared tests were used to assess heterogeneity across studies.

## Results

The main analyses of CPRD data included 9,498 breast cancer cases and 18,171 closely matched controls (Table 1). By design, cases and controls were the same age at index date (mean 43 [SD 5] years) and had identical observation periods (mean 7.3 [SD 4.6] years, range 1 to 22 years). Overall, 2% of the cases and controls were aged below 30 years, 21% were aged 30 to 39 years, and 77% aged 40 to 49 years. The characteristics of the cases and controls were similar, except that cases were somewhat more likely than controls to have had a recent birth recorded within the observation period; this was adjusted for in the main analyses.

**Table 1:** Characteristics of cases and controls in the CPRD data.

Characteristic	Cases *n* = 9,498	Controls *n* = 18,171
Observation period, mean years (SD)	7.3 (4.6)	7.3 (4.6)
Age at index date, N (%)		
20–29 years	174 (1.8)	345 (1.9)
30–39 years	1,941 (20.4)	3,844 (21.2)
40–49 years	7,383 (77.7)	13,982 (76.9)
One or more hormonal contraceptive prescription, N (%)	4,195 (44.1)	7,092 (39.0)
Number of recorded births, N (%)		
No recorded births	4,039 (42.5)	7,708 (42.4)
1–2 births	4,493 (47.3)	8,262 (45.5)
3+ births	996 (10.2)	2,201 (12.1)
Time since last recorded birth (years)^a^, N (%)		
< 5 years	897 (9.4)	1,357 (7.5)
5–10 years	713 (7.5)	1,064 (5.9)
No birth recorded in the last 10 years	7,888 (83.0)	15,750 (86.7)
BMI [kg/m^2^]^b^, N (%)		
<25	3,955 (52.5)	6,604 (47.9)
25+	3,579 (47.5)	7,171 (52.1)
Alcohol intake^b^, N (%)		
Nondrinker and past drinker	1,156 (16.7)	2,050 (16.9)
Drinker	5,772 (83.3)	10,088 (83.1)
Tubal ligation, N (%)	478 (5.0)	965 (5.3)
Hysterectomy, N (%)	331 (3.5)	622 (3.4)
Bilateral oophorectomy, N (%)	36 (0.4)	79 (0.4)

^a^Births during the observation period only.

^b^Percentages for alcohol intake and BMI are expressed as proportions of women with known values only. 23.0% of women had missing BMI (20.7% cases, 24.2% controls). 31.1% of women had missing data on alcohol consumption (27.0% cases, 33.2% controls).

BMI, body mass index; CPRD, Clinical Practice Research Datalink; SD, standard deviation.

During the observation period, 4,195 cases (44%) and 7,092 controls (39%) had one or more prescriptions for a hormonal contraceptive, and around two-thirds of these women (7,511/11,287; 67%) had been prescribed only one type of hormonal contraceptive during their observation window. Prescriptions varied considerably by age: for example, among 20- to 29-year-olds, 67% had received a prescription for any hormonal contraceptive in the previous 5 years (among whom the type last prescribed was: 77% oral combined, 12% oral progestagen-only, and 2% progestagen-releasing IUD); among 30- to 39-year-olds, 48% had received a prescription (60% oral combined, 23% oral progestagen-only, and 7% progestagen-releasing IUD); and among 40- to 49-year-olds, 25% had received a prescription (34% oral combined, 38% oral progestagen-only, and 17% progestagen-releasing IUD).

**Fig 1:**
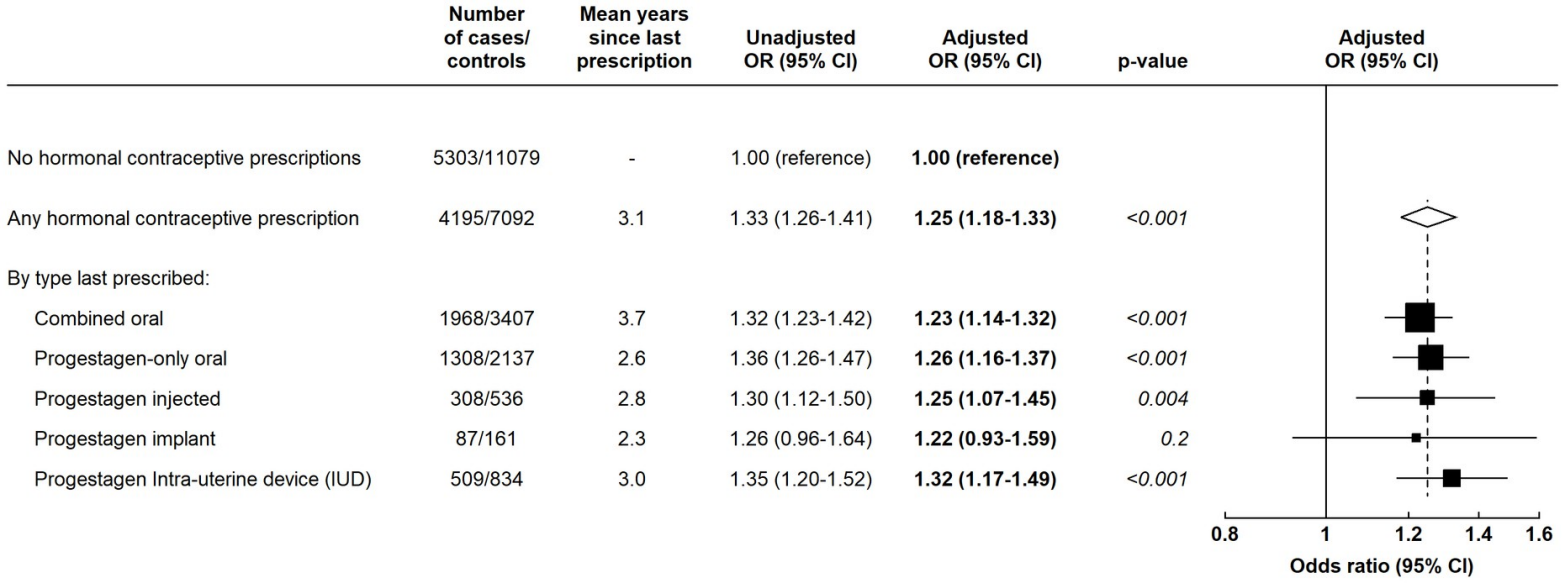
ORs and 95% CIs for breast cancer in women aged <50 years with any versus no prescriptions for hormonal contraceptives by the last type prescribed. Data from the CPRD. Adjusted ORs are adjusted for time since last birth, number of recorded births, BMI, and alcohol intake. *P* values are based on the relevant Wald tests. BMI, body mass index; CI, confidence interval; CPRD, Clinical Practice Research Datalink; IUD, intrauterine device; OR, odds ratio.

Compared to women with no hormonal contraceptive prescriptions during the observation period, women with at least 1 prescription had significantly increased odds of incident breast cancer (unadjusted OR = 1.33; 95% CI [1.26 to 1.41]; adjusted OR = 1.25; 95% CI [1.18 to 1.33]; *p* < 0.001). The mean time between the last hormonal contraceptive prescription and diagnosis of breast cancer (or equivalent date for controls) was 3.1 (SD 3.7) years. Fig 1 shows the OR for breast cancer associated with one or more prescriptions by the type of hormonal contraceptive last prescribed. All ORs were increased and did not vary by the type of hormonal contraceptive last prescribed (test for heterogeneity *p* = 0.9). For the oral combined, oral progestagen-only, injectable progestagen, progestagen implant, and progestagen IUD, the ORs were, respectively, 1.23 (95% CI [1.14 to 1.32]; *p* < 0.001), 1.26 (95% CI [1.16 to 1.37]; *p* < 0.001), 1.25 (95% CI [1.07 to 1.45]; *p* = 0.004), 1.22 (95% CI [0.93 to 1.59]; *p* = 0.2), and 1.32 (95% CI [1.17 to 1.49]; *p* < 0.001); every OR was significantly elevated, except for implanted progestagens, where the numbers were small and the CI correspondingly wide. To examine the extent to which these associations may have been affected by confounding with prior use of other types of hormonal contraceptives, we repeated this analysis among the 7,473 women with an observation period of at least 10 years, and restricting to women with no recorded use of any other hormonal contraceptive type within their observation period. The results were broadly similar for each type of hormonal contraceptive, respectively, with estimated ORs of 1.32 (95% CI [1.15 to 1.52]; *p* < 0.001), 1.35 (95% CI [1.09 to 1.65]; *p* = 0.005), 1.17 (95% CI [0.82 to 1.68]; *p* = 0.4), 1.39 (95% CI [0.55 to 3.52]; *p* = 0.7), and 1.40 (95% CI [1.04 to 1.87]; *p* = 0.03). All of these ORs were significantly elevated with the exception of those for injected progestagens and implanted progestagens, for which the numbers of exposed cases were extremely small (47 and 7, respectively), but there was no evidence that these ORs were materially different from the corresponding estimates from the main analysis.

**Fig 2:**
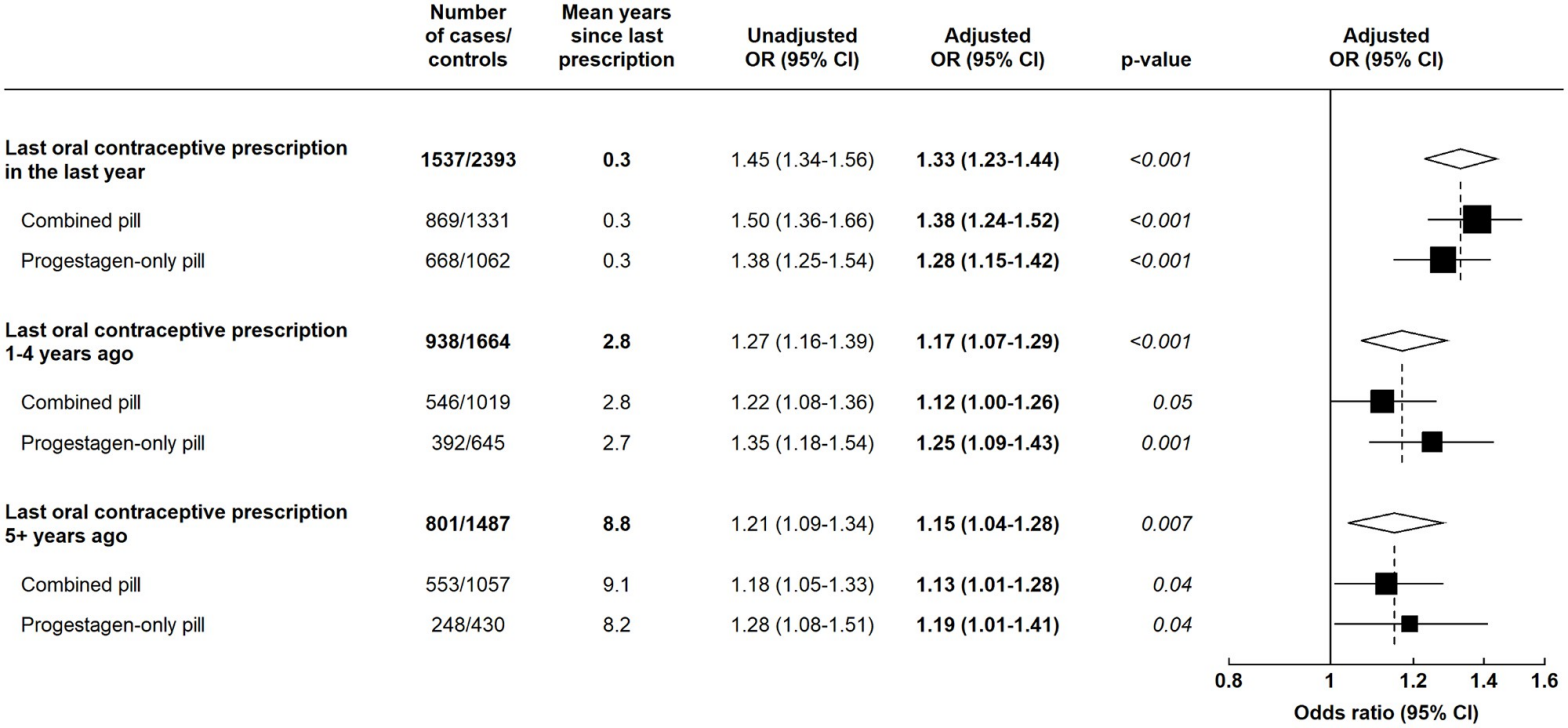
ORs and 95% CIs for breast cancer in women aged <50 years whose last hormonal contraceptive prescription was an oral contraceptive versus women with no prescriptions for hormonal contraceptives, by time since last prescription and oral contraceptive type. Data from the CPRD. Adjusted ORs are adjusted for time since last birth, number of recorded births, BMI, and alcohol intake. *P* values are based on the relevant Wald tests. BMI, body mass index; CI, confidence interval; CPRD, Clinical Practice Research Datalink; OR, odds ratio.

Oral contraceptives (either combined or progestagen-only) are effective only while they are being used, whereas injected, implanted, and intrauterine hormone-releasing contraceptives can be effective for months or even years [20]. To examine for any persistent effects after exposure to the hormones ceased, analyses focussed only on women who were last prescribed oral preparations as it is unclear when hormonal exposure would have ceased for those who last used nonoral preparations (Fig 2). Among current users of these oral preparations (among whom the last prescription was an average of 0.3 years prior to the index date), there was a 33% excess risk of breast cancer compared to women with no hormonal contraceptive prescription (OR = 1.33; 95% CI [1.23 to 1.44]; *p* < 0.001). The ORs declined by time since last use (test for heterogeneity *p* = 0.01), although only around a quarter of all cases had their last prescription more than 5 years previously. In every category of time since last use, ORs did not differ between users of oral combined and of oral progestagen-only contraceptives: for example, among the current users, the ORs were 1.38 (95% CI [1.24 to 1.52]; *p* < 0.001) and 1.28 (95% CI [1.15 to 1.42]; *p* < 0.001), respectively.

**Fig 3:**
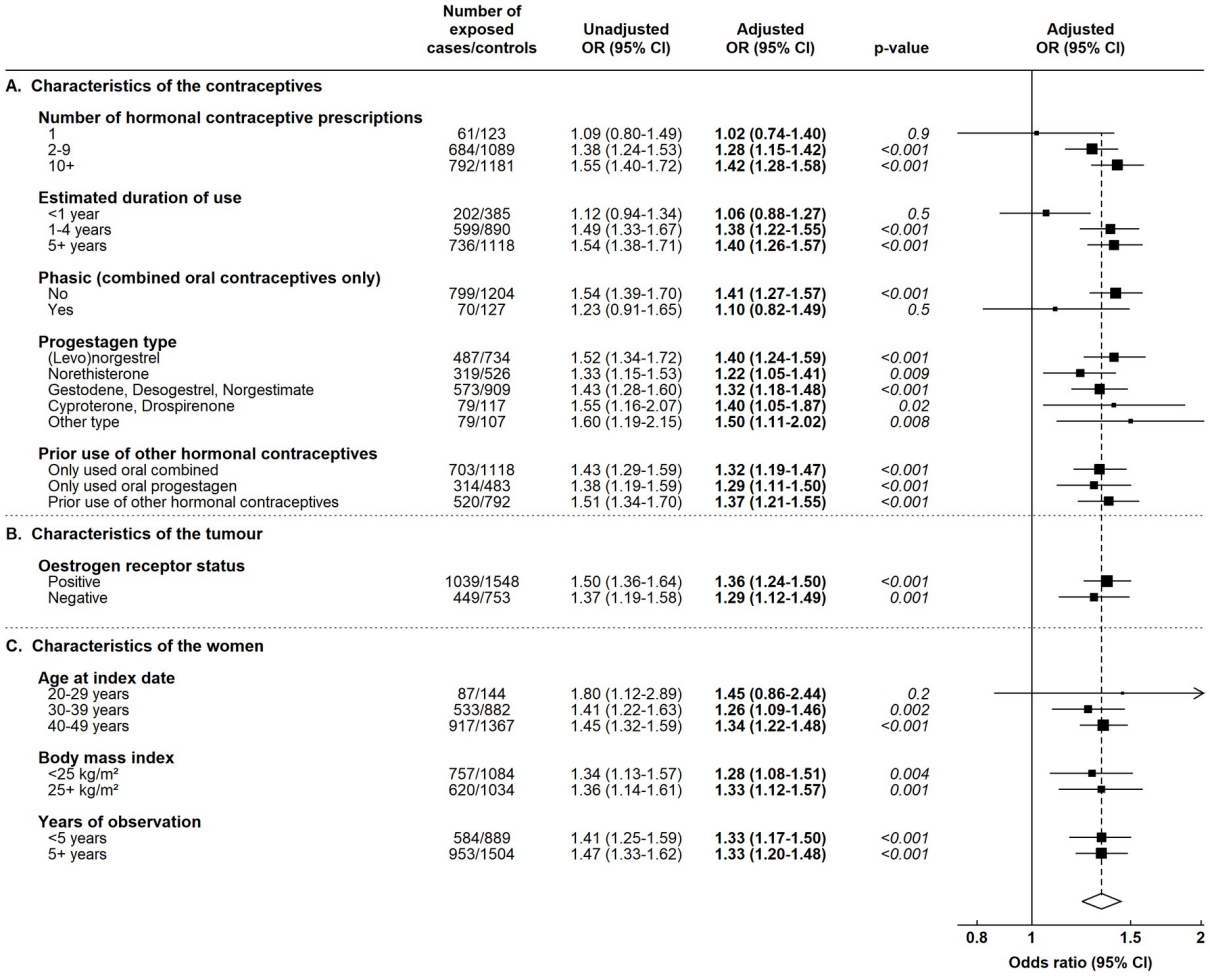
ORs and 95% CIs for breast cancer among current users of oral contraceptives versus women with no hormonal contraceptive prescriptions, across subgroups defined by characteristics of the oral contraceptives, breast tumour, or women. Data from the CPRD. Adjusted ORs are adjusted for time since last birth, number of recorded births, BMI, and alcohol intake. *P* values are based on the relevant Wald tests. BMI, body mass index; CI, confidence interval; CPRD, Clinical Practice Research Datalink; OR, odds ratio.

Fig 3 shows the ORs for breast cancer in current users of oral preparations in various subgroups. There was little effect of 1 year’s duration of use (*p* = 0.02 for 1 year versus longer durations) but no significant variation in the ORs between phasic and nonphasic formulations, by the progestagenic component of the preparations, by whether or not other hormonal contraceptive types had been used beforehand, by estimated ER status of the breast tumours, or across categories of women defined by their age or BMI.

**Fig 4:**
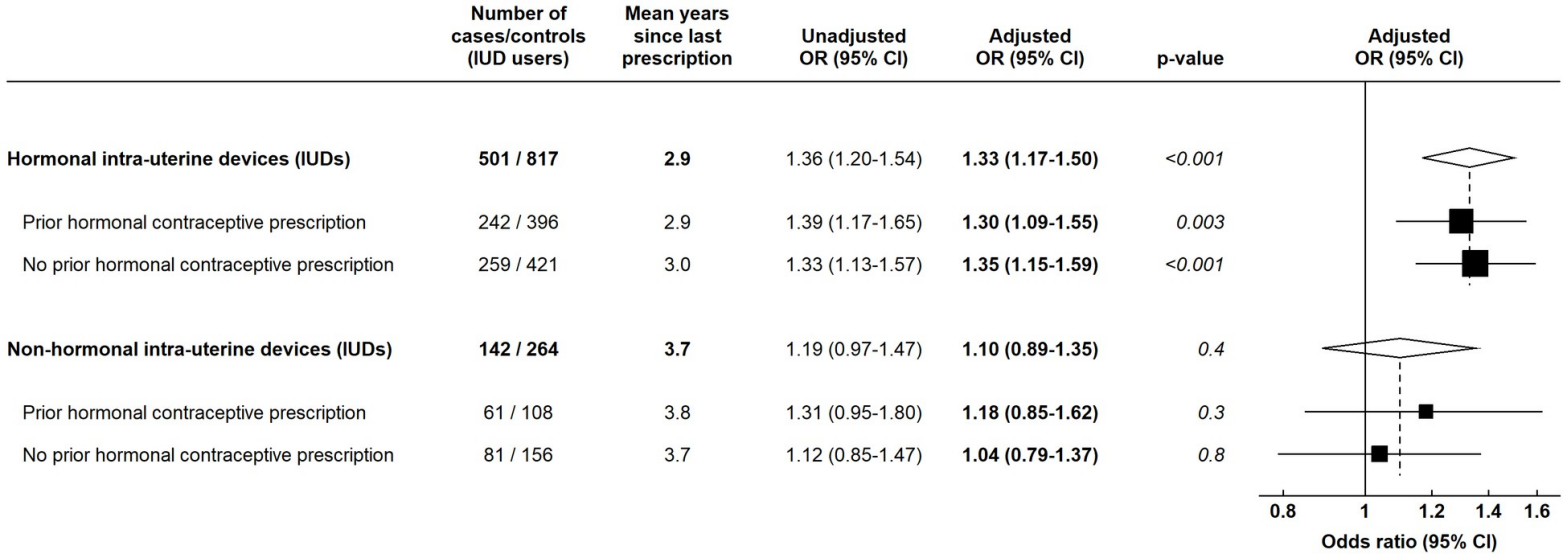
ORs and 95% CIs for breast cancer in women last prescribed an IUD versus women with no contraceptive prescriptions. Data from the CPRD. All ORs are versus women with no recorded prescriptions for either a hormonal contraceptive or nonhormonal IUD during the observation period. Numbers may vary from previous analyses as women whose last contraceptive prescription was for a nonhormonal IUD are considered under this category, even if they had previously received a hormonal contraceptive prescription. Adjusted ORs are adjusted for time since last birth, number of recorded births, BMI, and alcohol intake. *P* values are based on the relevant Wald tests. BMI, body mass index; CI, confidence interval; CPRD, Clinical Practice Research Datalink; IUD, intrauterine device; OR, odds ratio.

ORs for breast cancer were increased in women last prescribed a progestagen-releasing IUD (Fig 1). To investigate these findings further, we assessed whether this OR differed according to whether or not there was a previous prescription for other hormonal contraceptives and found no evidence of any difference in the magnitude of the effect (Fig 4; test for heterogeneity *p* = 0.8). We also examined whether use of nonhormonal (i.e., copper) IUDs was associated with breast cancer risk; the reference group for these analyses was women with no prescription for either a hormonal contraceptive or a nonhormonal IUD during the observation period. For women last prescribed nonhormonal IUDs, the OR for breast cancer was not significantly elevated (OR = 1.10; 95% CI [0.89 to 1.35]; *p* = 0.4, based on just 142 cases and 264 controls), although this OR was not significantly different from that associated with progestagen-releasing IUDs (OR = 1.33; 95% CI [1.17 to 1.50]; *p* < 0.001) (Fig 4).

The findings for those currently or recently prescribed any type of hormonal contraceptive were not materially altered in various sensitivity analyses: defining exposure as 2 or more hormonal contraceptive prescriptions; by restricting analyses to women with an observation period of more than 5 years; by restricting analyses to women with no missing records for any adjustment variables; or by excluding women with tubal ligation, hysterectomy, or bilateral salpingo-oophorectomy (S1 Table). Nor was breast cancer risk associated with other commonly repeated noncontraceptive prescriptions: nonsedating antihistamines (OR = 1.01; 95% CI [0.95 to 1.08]; *p* = 0.7), antibacterial eye preparations (OR = 1.04; 95% CI [0.97 to 1.11]; *p* = 0.3), or corticosteroids for asthma and other respiratory diseases (OR = 1.02; 95% CI [0.94 to 1.11]; *p* = 0.6) (S2 Table).

**Fig 5:**
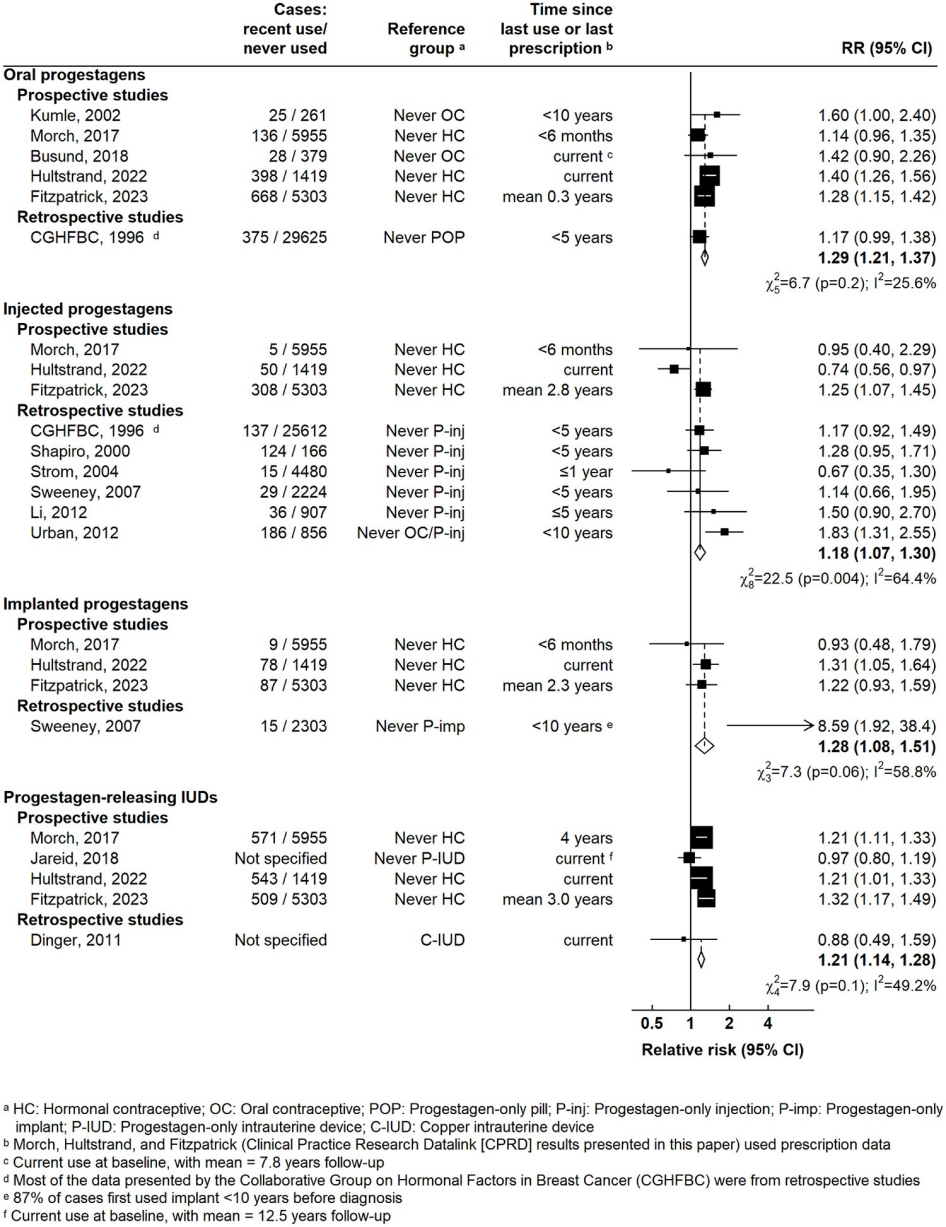
Meta-analysis of the RR for breast cancer associated with current or recent use of progestagen-only contraceptives. Results are presented separately for studies that recorded information prospectively, i.e., where information on contraceptive use was recorded prior to breast cancer diagnosis, and for studies that recorded information retrospectively. CI, confidence interval; RR, relative risk.

Our meta-analysis was restricted to evidence for progestagen-only contraceptives, as ample evidence already exists for oral combined contraceptives [1]. We identified 1 previous meta-analysis and 11 other eligible studies (S1 Fig and S3 Table) [1–12]. Combining published results with the new results from CPRD yielded significant excess risks (*p* < 0.001) for all 4 preparation types (Fig 5): oral progestagen-only contraceptives (RR = 1.29; 95% CI [1.21 to 1.37]; heterogeneity χ^2^_5_ = 6.7; *p* = 0.2), injected progestagens (RR = 1.18; 95% CI [1.07 to 1.30]; heterogeneity χ^2^_8_ = 22.5; *p* = 0.004), implanted progestagens (RR = 1.28; 95% CI [1.08 to 1.51]; heterogeneity χ^2^_3_ = 7.3; *p* = 0.06), and progestagen-releasing IUDs (RR = 1.21; 95% CI [1.14 to 1.28]; heterogeneity χ^2^_4_ = 7.9; *p* = 0.1). There was no heterogeneity between the RRs for the 4 types of progestagen-only contraceptives (test for heterogeneity *p* = 0.3). Results were similar when restricted to studies that only included premenopausal women, to studies with prospectively recorded information, and to studies where women in the reference group had used neither progestagen-only nor combined oral contraceptives (S2 Fig). There was no evidence of publication bias based on funnel plots (S3 Fig).

**Fig 6:**
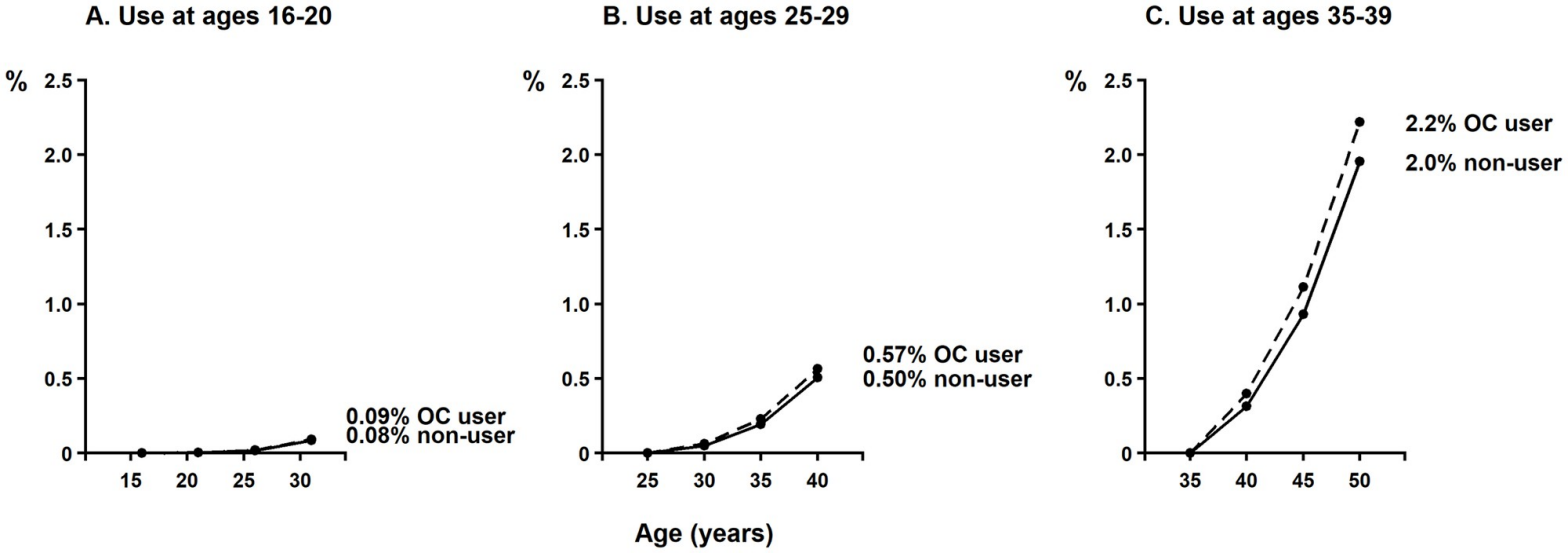
Absolute risk (%) of breast cancer over a 15-year period associated with 5 years use of OCs at different ages. Absolute risks include the excess risks in current users during the 5 years when the OC is used and the excess risks in the 10 years after stopping. There is no excess risk more than 10 years after stopping. OC, oral contraceptive.

Combining the CPRD results on time since last use of oral contraceptives, be they combined or progestagen-only, with previously published findings [1] yielded RRs of 1.27 (95% CI [1.21 to 1.33]) for current use; 1.16 (95% CI [1.11 to 1.22]) for last use 1 to 4 years ago; 1.08 (95% CI [1.04 to 1.13]) for last use 5 to 9 years ago; with no excess risk 10 or more years after stopping use. Based on these RR estimates, the absolute excess incidence of breast cancer among women in Western countries who use oral combined or progestagen-only contraceptives for 5 years can be estimated (S4 Table). These estimates are of absolute risk over a 15-year period after starting oral contraceptive use and include both the excess risks during 5 years of current use and the excess risks during the 10 years after use stopped. Breast cancer incidence in nonusers is extremely rare before about age 30 and increases sharply with age thereafter. This is reflected in the estimated absolute excess incidence (S4 Table), where the 15-year excess absolute risk of breast cancer for use at ages 16 to 20 years is about 8 per 100,000 users (an increase in incidence from 0.084% to 0.093%); for use at ages 25 to 29 is about 61 per 100,000 users (from 0.50% to 0.57%); and for use at ages 35 to 39 is about 265 per 100,000 users (from 2.0% to 2.2%). Fig 6 shows the estimated age-specific absolute risks associated with hormonal contraceptive use at these ages.

## Discussion

In a large nested case–control study, which included almost 10,000 UK women aged <50 years with breast cancer, those prescribed oral combined contraceptives (containing oestrogen+progestagen), oral progestagen-only contraceptives, injectable progestagens, and progestagen-releasing IUD contraceptives were found to be at increased risk of breast cancer. The ORs for each of these hormonal contraceptives were statistically significant but comparatively small, at around 1.2 to 1.3, with no material difference between the different hormonal contraceptive types. The average time between the last prescription and breast cancer diagnosis was about 3 years, so these results generally apply to current or recent use of these hormonal preparations.

The ORs for breast cancer among current or recent users of each of these hormonal contraceptives were of similar magnitude to previously reported risks associated with use of oral combined oestrogen-progestagen contraceptives [1]. Fewer studies have published on the risks associated with progestagen-only contraceptive use, however, and so our meta-analysis aimed to bring together the totality of the available evidence. In the meta-analysis, breast cancer risks were similarly elevated among current or recent users of oral progestagens, injectable progestagens, progestagen implants, and progestagen-releasing IUDs, with respective RRs of 1.29, 1.18, 1.28, and 1.21. Every RR was significantly elevated (*p* < 0.005), although with substantial heterogeneity in risks for injected progestagens.

Doses of oestrogen and progestagen constituents in combined oral contraceptives are generally lower than they were in previous decades [21]. Although preparations used by women in the CPRD data are likely to have been of lower dose, on average, than those used by women in the previously published meta-analysis [1], findings from both studies are consistent. One puzzling finding in the current analysis, however, is the excess breast cancer risk associated with use of progestagen-releasing contraceptive IUDs, which is of similar magnitude to the excess risks found for oral and for other parenteral progestagens. This excess did not appear to be due to prior prescriptions for other hormonal contraceptives and is also consistent with the only other published prospective evidence restricted to premenopausal women [10,12]. Phase II and III trials and pharmacokinetic analyses suggest that serum levonorgestrel levels associated with levonorgestrel-releasing IUDs are considerably lower than the levels associated with other levonorgestrel-containing oral or parenteral contraceptives [22–25]. We attempted to investigate whether breast cancer risk associated with use of hormonal IUDs was greater than with use of nonhormonal IUDs, but too few women had been prescribed nonhormonal IUDs for reliable comparison.

We considered the possibility that breast cancers might be selectively diagnosed among women who regularly seek prescriptions from their GPs. Any such selective detection might be expected to be greater for oral preparations that require more frequent prescriptions than for long-acting preparations (such as IUDs), but no such differences were found. Further evidence against possible detection bias is that no excess breast cancer risk was associated with other common prescriptions that are often repeated (for antihistamines, antibacterial eye preparations, or corticosteroids for asthma and other respiratory disease).

A major strength of the CPRD analysis presented here is that information on hormonal contraceptive prescribing was recorded prospectively, with reliable information on specific preparations, thus avoiding bias associated with selective recall of contraceptive use after breast cancer has been diagnosed, and misclassification of exposures due to reporting errors. The analyses of CPRD data presented here were matched on GP practice, providing some degree of adjustment for socioeconomic status, and were also adjusted for established risk factors for breast cancer, which might be expected to confound the association between contraceptive use and breast cancer risk. The only exception to this was family history of breast cancer, as information on this factor was relatively incomplete, particularly for controls, as this information was often only recorded around the time of breast cancer diagnosis. While it is unclear what effect, if any, adjustment for family history of breast cancer would have made to our findings, previously published findings for combined oral contraceptives [26] were unaltered after adjustment for family history, and 2 studies of progestagen-only contraceptives included in our meta-analysis [3,11] found little change in associated risks after adjustment for a number of additional factors including family history. Data on BMI and alcohol use were missing for some women, but sensitivity analyses restricted to those with complete data yielded similar results. Although information on earlier births may be missing for some women, information on recent births, which are more likely to confound any association of recent contraceptive use with breast cancer risk, should be relatively complete. To avoid potential biases associated with the menopause, analyses were restricted to cases and controls younger than 50 years, excluding women with prescriptions for menopausal hormonal therapy.

A limitation of the CPRD data, shared with some other prescription databases, is that the prescriptions are recorded during a defined period only, with information before entry into such databases generally being unavailable. While a lack of complete prescription data makes it difficult to assess the long-term effects of contraceptive use, it does not unduly affect estimates of the short-term effects of such use, which is the main focus of these investigations. The lack of information on hormonal contraceptive use prior to the start of the observation period also means we were unable to allow for the effect of possible differences in duration of use of the different types of hormonal contraceptives on their relative associations with breast cancer risk. Since our analyses of risks associated with specific types of contraceptives categorised women according to the preparation last prescribed, it is possible that prior patterns of contraceptive use could have confounded associations of breast cancer risk with type of preparation last prescribed. However, when we examined risks in women with an observation period of at least 10 years and no prior use of other types of hormonal contraceptives, the results were similar, suggesting that any such confounding had little effect on our results. It is possible that some hormonal contraceptives were prescribed outside UK general practices, but this is uncommon and would, if anything, attenuate any estimated ORs. Furthermore, although some women may not fill the prescriptions they received, those with repeated prescriptions would be more likely to have done so, and their ORs were similar to those in the main findings. While use of primary care data, as opposed to cancer registration data, for ascertainment of breast cancer cases may have led to a small degree of misclassification with respect to breast cancer status, a recent validation study found that the vast majority of breast cancers identified through primary care data (approximately 96%) can be verified through cancer registry data [27].

Perhaps the greatest potential source of bias in the findings from studies included in our meta-analysis is that due to recall bias in studies where information on hormonal contraceptives was recorded after a diagnosis of breast cancer. However, restriction of the meta-analysis to studies with prospectively collected information on contraceptive use yielded similar results, and so recall bias is unlikely to have had a major impact on our findings. Inadequate adjustment for important confounders could also have biased the results of certain studies, but in those studies that assessed the impact of adjustment for other factors in addition to age, ethnicity, and sociodemographic status (S3 Table), it appeared to have little impact on the results, suggesting that residual confounding by other risk factors has not unduly affected the findings. There is likely to be some degree of misclassification in the recording of hormonal contraceptive use, particularly in those studies that rely on self-reported information, but this should not have materially affected the findings since the 3 largest studies that contributed to the meta-analysis were based on prescription records. We found no good evidence of publication bias based on funnel plots for each association of interest (S3 Fig), although there is likely to be limited power to detect any such bias given the small number of studies included.

The excess RRs for breast cancer appear to be of similar magnitude in current or recent users of combined oestrogen-progestagen and of progestagen-only contraceptives, be they orally or parenterally administered. In nonusers of hormonal contraceptives, breast cancer incidence is extremely rare before age 30 but increases sharply with age. The estimated absolute excess incidence of breast cancer in current or recent users of oral contraceptives is, therefore, much smaller for use at younger than at older ages (for example, for women in high-income countries, the excess breast cancer incidence would be about 8 per 100,000 for use at age 16 to 20 years, but 265 per 100,000 for use at age 35 to 39 years). Given that the RRs are similar for oral preparations, injected progestagens, implanted progestagens and progestagen-releasing IUDs, these estimated absolute excess risks would be broadly similar for all types of hormonal contraceptives. These results, which are based on the combination of data from a large number of worldwide studies, are expected to be generalisable to other populations; however, breast cancer incidence is lower in middle-income and low-income than in high-income countries, and, thus, the absolute excess risks would also be expected to be lower. These risks need, of course, to be considered in the context of the benefits of contraceptive use in the childbearing years.

The mechanisms underlying the effects of progestagens on the development of breast cancer are poorly understood. It is clear that among postmenopausal women, breast cancer RRs are considerably greater with use of hormonal therapies containing both progestagens and oestrogens than oestrogens alone [28]. However, menopausal hormone therapies are usually taken during a period when ovarian function has ceased, and endogenous oestrogen and progesterone levels are relatively low, whereas oral contraceptives are taken during the reproductive years when levels of these hormones are much higher, and so their likely impact on overall exposure to hormones during use is less clear. Risks associated with hormonal contraceptive use did not appear to differ by estimated ER status (based on the presence or absence of a Tamoxifen prescription in the months after diagnosis), whereas a clear excess of ER-positive tumours was found for menopausal hormone therapy [28]. Further research is therefore needed to elucidate the mechanisms behind the similar associations of recent use of combined and progestagen-only contraceptives with breast cancer risk observed here.

## Supporting information

S1 STROBE Checklist STROBE checklist. (DOCX)

S1 PRISMA Checklist PRISMA checklist. (DOCX)

S1 Protocol ISAC protocol. (DOCX)

S1 File Additional information about CPRD and list of breast cancer Read codes. (DOCX)

S2 File Breast cancer risk per 100,000 never users of hormonal contraceptives. (DOCX)

S1 Table Sensitivity analyses. (DOCX)

S2 Table ORs for breast cancer associated with use of other medications. (DOCX)

S3 Table Summary of studies included in meta-analyses. (DOCX)

S4 Table Estimated excess incidence of breast cancer per 100,000 women. (DOCX)

S1 Fig PRISMA flow diagram. (DOCX)

S2 Fig Sensitivity analyses for meta-analyses. (DOCX)

S3 Fig Funnel plots for assessment of publication bias. (DOCX)

## Abbreviations

BMI: body mass index
CI: confidence interval
CPRD: Clinical Practice Research Datalink
ER: oestrogen-receptor
GP: general practitioner
IUD: intrauterine device
NHS: National Health Service
OR: odds ratio
RR: relative risk
SD: standard deviation
