# Supplementary material for: Combined and progestagen-only hormonal contraceptives and breast cancer risk: A UK nested case–control study and meta-analysis
Source: PLoS Med. 2023 Mar 21;20(3):e1004188. doi: 10.1371/journal.pmed.1004188 (PMC10030023; doi:10.1371/journal.pmed.1004188)
Supplement: S1 Protocol — (DOCX) [file pmed.1004188.s003.docx]

ISAC APPLICATION FORM:

PROTOCOLS FOR RESEARCH USING THE GPRD DATA

| ISAC use only:  Protocol Number  Date submitted | Team 2  10_152  26/10/2010 | **IMPORTANT**  **If you have any queries, please contact ISAC Secretariat: ISAC**[@gprd.com](mailto:Annalisa.Rubino@gprd.com) |
| --- | --- | --- |

| 1. Study Title        Risk of breast cancer in users of exogenous female hormones |
| --- |

| 1. Does this protocol describe a purely observational study using GPRD data (this may include the review of anonymised free text)?   Yes xNo |
| --- |

| 1. Does this protocol also seek access to data held under the GPRD Data Linkage Scheme?   Yes No x |
| --- |
| 1. If you are seeking access to data held under the GPRD Data Linkage Scheme, please select the source/s of linked data being requested.   Hospital Episode Statistic^†^  Cancer Registry Data *  MINAP*  Townsend Score  ONS Mortality Data  Other: *(please specify)*  ** Please note that access to these data sources are not covered under the GPRD-MRC Scheme.*  ^†^ *Please note that only limited access to this data source is covered under the GPRD-MRC Scheme.* |
| 1. If you are seeking access to data held under the GPRD Data Linkage Scheme, have you discussed your request   with a member of the Research team?  Yes  No  If *No*, please contact the GPRD Research Team to discuss your requirements before submitting your application. |

| 1. Does this protocol involve requesting any additional information from GPs?   Yes No x  If yes, please indicate what will be required:    Completion of questionnaires by the GP* Yes No  Provision of anonymised records (e.g. hospital discharge summaries) Yes No  Other (please describe)  *any questionnaire for completion by GPs needs to be approved by ISAC before being sent out for completion. |
| --- |

**GUIDANCE ON ANSWERING QUESTIONS 4-6:**

**These questions must be completed by all applicants. You should note the following:**

**(i) If you have answered NO to question 2, may need to seek separate ethics approval from an NHS Research Ethics Committee for this study. The ISAC will provide advice on whether this may be needed.**

**(ii) If you have answered YES answered to question 2 above and you will be using data obtained from the GPRD Group at the MHRA, this study does not require separate ethics approval from an NHS Research Ethics Committee.**

**If you will be using data obtained from EPIC, you will need to consult the data provider regarding their arrangements for obtaining ethics approval for the study.**

**NB:** Answering YES to question 2 means that the answers to questions 7-9 should all be NO. If any of the answers below are YES please review your answer to question 2 as it should be NO.

| 1. Has this protocol been peer reviewed by another Committee? Yes No x   *If yes, please state in your protocol the name of the reviewing Committee/s and provide an outline of the*  *review process and outcome on final review.* |
| --- |

| 1. Does the study involve linking to patient *identifiable* data from other sources? Yes No x |
| --- |

| 1. Does this study require contact with patients in order for them to   complete a questionnaire? Yes No x |
| --- |

| 1. Does this study require contact with patients in order to collect a sample? Yes No x   If yes, please state what will be collected |
| --- |

| 1. Type of Study *(please tick one box below)*   Adverse Drug Reaction Drug Use Disease Epidemiology xPharmacoeconomic Drug Effectiveness  Other |
| --- |

| 1. Data source *(please tick one box below)*   GPRD Division at MHRA Other  *(please specify)*  Full Feature on-line access  Ad hoc dataset  MRC dataset  *x*  Other commissioned study |
| --- |

| 1. Financial Sponsor of study   MRC* xPharmaceutical Industry *(please specify)*  Government / NHS *(please specify)*       Other *(please specify)*  None  * Tick this box if you wish to access GPRD data under the MRC licence with GPRD. It is expected that if you use the MRC licence, no other direct commercial/public sector funding for this study will be sought/has been applied for or is in place. If funding is in place, but does not cover the use or extraction of GPRD data, please tick the boxes for relevant funding sources (including MRC) and provide details in the protocol of why funding under the MRC licence is required. |
| --- |

| 1. Is the study intended for   Publication in peer reviewed journals xPresentation at scientific conference x  Presentation at company/institutional meetings Other |
| --- |

| 1. Principal Investigator (full name, job title, organisation & e-mail address for correspondence regarding this protocol)   Dr Jane Green  Clinical Epidemiologist  University of Oxford  jane.green@ceu.ox.ac.uk |
| --- |

| 1. Affiliation (full address)        Cancer Epidemiology Unit  Nuffield department of Clinical Medicine  University of Oxford  Richard Doll Building  Roosevelt Drive  Oxford OX3 7LF |
| --- |

| 1. Type of Institution *(please tick one box below)*   Academia xResearch Service Provider Pharmaceutical Industry  NHS Government Departments Others |
| --- |

| 1. Experience/expertise available   Please complete the following questions to indicate the experience/expertise available within the team of researchers actively involved in the proposed research, including analysis of data and interpretation of results |
| --- |
| Previous GPRD Publications  Studies using GPRD data  None  1-3 x **x**  > 3 |
| Yes No  Is statistical expertise available within the research team? x  *If yes, please outline level of experience extensive, including GPRD analyses*  Is experience of handling large data sets (>1 million records) x  available within the research team?  *If yes, please outline level of experience extensive; Million women Study cohort, GPRD*  Is UK primary care experience available within the research team? x  *If yes, please outline level of experience no current direct experience but 2 proposers have clinical qualifications and advice available from University department of primary care.* |

| 1. Other collaborators (if applicable: *please list names and affiliations of all collaborators*) |
| --- |

| 1. Protocol’s Author (if different from PI) |
| --- |

**Protocol content checklist**

In order to help ensure that protocols submitted for review contain adequate information for protocol evaluation, ISAC have produced instructions on the content of protocols for research using GPRD data. These instructions are available on the GPRD website ([www.gprd.com/ISAC](http://www.gprd.com/ISAC)). All protocols using GPRD data which are submitted for review by ISAC must contain information on the areas detailed in the instructions. IF you do not feel that a specific area required by ISAC is relevant for your protocol, you will need to justify this decision to ISAC.

Applicants must complete the checklist below to confirm that the protocol being submitted includes all the areas required by ISAC, or to provide justification where a required area is not considered to be relevant for a specific protocol. Protocols will not be circulated to ISAC for review until the checklist has been completed by the applicant.

**Please note, your protocol will be returned to you if you do not complete this checklist, or if you answer ‘no’ and fail to include justification for the omission of any required area.**

|  | **Included in protocol?** | |  |
| --- | --- | --- | --- |
| **Required area** | **Yes** | **No** | **If no, reason for omission** |
| ***Lay Summary (max.200 words)*** | **x** |  |  |
| ***Objective, specific aims and rationale*** | **x** |  |  |
| ***Background*** | **x** |  |  |
| ***Study Type***  *Hypothesis Generating*  *Hypothesis Testing* | **x** |  |  |
| ***Study Design Methodology*** | **x** |  |  |
| ***Study population, including estimate of expected number of relevant patients in the GPRD*** | **x** |  |  |
| ***Sample size/ power calculation*** | **x** |  | *Please provide detailed justification of sample size in the protocol* |
| ***Selection of comparison group(s) or controls*** | **x** |  |  |
| ***Exposures, outcomes and covariates*** | **x** |  |  |
| ***Data/ Statistical Analysis***  *Hypothesis Generating*  *Hypothesis Testing* | **x** |  |  |
| ***Patient/ user group involvement ^†^*** |  | **x** | **not necessary for this analysis** |
| ***Limitations of the study design, data sources and analytic methods*** | **x** |  |  |
| ***Plans for disseminating and communicating study results*** | **x** |  |  |

***^†^ It is expected that many studies will benefit from the involvement of patient or user groups in their planning and refinement, and/or in the interpretation of the results and plans for further work. This is particularly, but not exclusively true of studies with interests in the impact on quality of life. Please indicate whether or not you intend to engage patients in any of the ways mentioned above.***

*ISAC strongly recommends that researchers using GPRD consider registering as a NRR data provider in order that others engaged in research within the UK can be made aware of current works. The* ***National Research Register (NRR)*** *is a register of ongoing and recently completed research projects funded by, or of interest to, the United Kingdom's National Health Service. Information on the NRR is available on* [*www.nrr.nhs.uk*](http://www.nrr.nhs.uk/) *.*

***Please Note: Registration with the NRR is entirely voluntary and will not replace information on ISAC approved protocols that are published in summary minutes or in the ISAC annual report****.*

Risk of incident breast cancer in users of exogenous female sex hormones (for contraception and hormone replacement therapy)

Cancer Epidemiology Unit, University of Oxford

Jane Green, Valerie Beral, Gill Reeves, Joanna Watson

Department of Statistics, University of Warwick

Gabriela Czanner

Lay summary of research

Breast cancer is strongly influenced by female sex hormones such as oestrogen and progesterone; both by those produced naturally in a woman's body (endogenous hormones), and by hormones taken for contraception or as hormone therapy for the menopause (HT) (exogenous hormones). Current users of both hormonal contraceptives and HT have an increased risk of breast cancer, up to 2-fold in the case of HT preparations containing both oestrogen and a progestagen (synthetic progesterone). Understanding these risks has direct implications for public health: for example, rates of breast cancer have fallen in response to falls in prescribing of HT, following publication of reliable evidence on breast cancer risk in recent years. There are however some gaps in our knowledge. It has been suggested that the effect of HT on breast cancer risk may depend on the timing of use (in relation to age or menopausal status); and there is limited information on the risks associated with progestagen-only contraceptives, or with specific contraceptive formulations. The GPRD's large size and the availability of detailed prescription data by type will allow us to examine these questions; our analyses would extend and supplement previous GPRD studies of hormone use and breast cancer.

Objectives, specific aims and rationale

*Research objective:* to obtain relative risks for incident breast cancer in relation to prior prescribing of contraceptive hormones and of hormone replacement therapy for the menopause (HT), by type and timing of use.

*Specific aims:* to add to existing knowledge about the effect of use of exogenous female sex hormones on risk of breast cancer, examining unanswered questions including the influence on risk of the timing of HT use, and suggested differences in risk associated with specific contraceptive preparations.

*Rationale:* current use of both HT and contraceptive hormones has been shown to increase risk of breast cancer, and this knowledge has direct public health implications. The large size and reliable prescribing information in the GPRD will allow us to add to existing knowledge by examining breast cancer risk in relation to specific hormone preparations and to timing of HT use, where there is at present suggestive but unconfirmed evidence for differences in risk.

Background

The development of breast cancer in women is strongly influenced by female sex hormones, and the risks associated with reproductive patterns (parity, age at first birth) and with use of oral contraceptives and of hormone therapy for the menopause have been extensively described [1-6]. However, unanswered questions remain. For hormonal contraceptives, there is good evidence for a modest increase in risk of breast cancer in current users of oral contraceptives containing both oestrogen and progestagen, both for older [4] and more recent [7] preparations, with relative risks for current compared with never use of around 1.3. There may be higher risks associated with specific formulations and modes of delivery, such as triphasic pills containing levonorgestrel [7], but evidence is limited. Similarly the risks associated with use of progestagen-only contraceptives, including the intra-uterine device Mirena, are not clear [4].

Some types at least of hormone therapy for the menopause are known to be associated with increased risk of breast cancer, with relative risks of about 2 reported for current use of combined oestrogen-progestagen preparations [8]. It has recently been suggested that the effect of HT use on breast cancer risk may vary depending on women's age and/or menopausal status when use starts, with risk higher for use starting around the time of the menopause than for later use [9]. Such findings have important potential implications for clinical practice and need to be investigated in independent datasets. There is debate too about the relative risks associated with different formulations of HT, including, again, the use of Mirena progestagen-releasing intrauterine devices [10]; as well as differences by mode of drug delivery (transdermal vs oral) [11].

We are not aware of previous research studies in the GPRD examining cancer risk in relation to use of hormonal contraceptives. For HT, five previous publications have described analyses of HT use and risk of breast cancer from the GPRD [12-15]. The most comprehensive, with 6,347 cases aged 50-75 [14], used data up to 2004; the most recent (1261 cases with data to 2007) focussed on risk associated with a specific progestagen, dydrogesterone [15]. Our proposal would allow updating and extension of the previous analyses to 2010, to include all HT types, including Mirena, and to examine risk by timing of use.

Reliable analyses by hormone type require large numbers of exposed cases, as well as detailed prospective information on prescribing. The GPRD has both, and sufficient information to allow adjustment or stratification by known potential confounders such as body mass index and alcohol use (as well as some less well-established risk factors for breast cancer such as smoking and use of aspirin).

Finally, a major source of evidence on the effects of exogenous sex hormones on breast cancer risk has been, and will continue to be, the results from pooled reanalyses of individual data included in the international Collaborative Group on Hormonal Factors in Breast Cancer mentioned above. The proposers of the current study also run this collaboration; collaborative results are currently being updated for both hormonal contraceptives and for hormone therapy for the menopause, and a comprehensive and up to date analysis of GPRD data would allow the optimal contribution from GPRD to the worldwide evidence.

Study type

Hypothesis testing and confirmation of findings of public health significance.

Study design

Nested case-control study within defined cohort in GPRD, using cases and controls matched for age, practice and observation time in the GPRD. 2 controls will be selected per case to maximise study power.

Study population

Women aged 20-79 years registered with GPRD on or after 1 January 1995.

Inclusion criteria:

- Age 20-79 [age group includes almost all cancers and hormone prescriptions]
- Registered with GPRD up –to-standard practice on or after 1 January 1995
- Fulfilling GPRD acceptable patient criteria
- At least 1 year in GPRD before index date (date of diagnosis of case)

Exclusion criteria:

- Previous invasive or *in situ* breast cancer

Case selection

Cases will be identified as any eligible patient with an incident diagnosis of invasive or *in situ* cancer of the breast between 1 January 1995 and latest available date.

Selection of comparison group (controls)

2 controls per case selected from eligible patients who do not have a medical diagnosis of breast cancer documented at any time up to the latest available date (above). This includes diagnoses of breast cancer prior to or during the study period. Controls and cases matched on age (within 2 years), practice and observation time in GPRD (ie controls to have observation time in GPRD at least as long as their matched case, to allow exact matching in the analysis; for cases, start date of observation = most recent of patient registration date, practice up-to-standard date and practice management start date, end date (index date) = date of diagnosis). Index date for controls defined as the diagnosis date of their matched case.

Sample size and power calculations

Incidence of invasive breast cancer in GPRD is similar to that in the general UK population, which is ~63 per 100,000 per year (Office for National Statistics (ONS), 2007) ie 630 cases per million person years. There are currently 62 million person years of research acceptable followup in GPRD (GPRD Gold October 2010 release), so we might expect some 39,000 incident breast cancers in the whole database, and of these about 20,000 will be in women aged 50-75 (ONS, 2007). It seems reasonable to assume, allowing for some restriction of age group and observation time, that our analyses would include at least 10,000 breast cancer cases and 40,000 controls. These numbers are consistent with those in the GPRD study by Opatrny et al, which included incident cases of invasive breast cancer in women aged 50-75 diagnosed between 1998 and 2004, and had over 6,000 cases [14]. From the Opatrny study and from our previous analyses in GPRD (protocol no. 10_006; analyses not yet published) the prevalence of HT prescribing in women over 40 years will be approximately 33 % (possibly lower in very recent years); OC prescribing in women 20-50 years is estimated at about 20 % in GPRD in recent studies of outcomes other than cancer [16].

With these numbers the study will be amply powered with 80% power and 95% confidence to detect a minimum relative risk of <1.1 in relation to overall risk for HT or OC use v no use, and of <=1.5 for any subgroup with exposure prevalence of 1% or more.

Exposures, outcomes, covariates

Exposure: patients will be considered exposed to exogenous hormones if they have at least one prescription for a hormonal contraceptive or hormone replacement therapy preparation (as defined in the British National Formulary (BNF)) within the observation period (start of follow-up to index date). Sensitivity analyses will be performed restricting the definition of exposure to those with 2 or more prescriptions. OC/HT type will be assigned based on BNF classification, classing women with separate prescriptions on the same day for oestrogen and progestagen as being prescribed combined oestrogen-progestagen therapy.

Outcomes: incident invasive and *in situ* cancers of the breast (ICD-10 C50 and D05)

Covariates: smoking status, alcohol drinking [most recent record before index date], body mass index [most recent record at least 2 years before index date, to account for reverse causality] and parity will be taken into account where possible in adjusted and/or stratified analyses. Availability of prescription data will allow medications such as aspirin and bisphosphonates, which may be related to breast cancer risk and to hormone use, to be considered in supplementary analyses.

Data/statistical analysis outline methodology

Conditional logistic regression will be used to estimate crude and adjusted odds ratios (relative risks) with 95% confidence intervals for cancer risk in relation to hormone prescription vs no prescription within the observation period. Analyses for hormonal contraceptives will include hormone type and mode of delivery, including progestagen-only contraception; for HT, hormone type, mode of delivery and timing of use; for both, the risks associated with use of Mirena will be examined. Adjustment variables will include smoking status (never, current, past, unknown), alcohol drinking (none, some, unknown) and body mass index (<25, 25-30, 30+ kg/m sq, unknown) and where possible, parity (at least nulliparous, parous, unknown). Data analysis will be carried out using STATA software v10. Analyses for HT will generally be restricted to women aged over 40, and for OC use to women aged 20-50 years.

Patient/user group involvement

None

Limitations of study design, data sources, analytic methods

Validation studies have shown GPRD outcome and exposure (prescription) data to be sufficiently reliable and complete for epidemiological analysis; and the GPRD population is sufficiently representative of the UK population for the results to be broadly generalisable.

Potential limitations of the GPRD for these analyses include the lack of complete information on smoking, alcohol and body mass index, and particularly on reproductive factors such as parity, breastfeeding history and age at first birth; and the fact that information on prescribing of hormonal contraceptives is likely to be incomplete, as prescriptions issued outside general practice will not always be recorded. It is estimated that 80% of all prescriptions for hormonal contraceptives in the UK are issued in general practice (UK Family Planning Organisation, personal communication). Data on other potential confounders, such as diet and socioeconomic status, are not available (the impact of the latter will to some extent be minimised by matching of cases and controls by GP practice).

Such missing data will have the effect of diluting the study power, but should not introduce bias unless data is missing for reasons related to the outcome. The consistency of the results of previous HT studies in GPRD with those from studies in which reproductive factors were taken into account argues against substantial bias in the database; similar comparisons will help in interpreting results on hormonal contraceptive use. Sensitivity analyses with restriction to patients with full data on adjustment variables (complete case analysis) will be performed to test the effect of missing data in the main analyses.

Use of other prescribed drugs which may affect breast cancer risk (eg bisphosphonates, NSAIDs) cannot readily be taken into account by adjustment as there are complex possible relationships between prescribing of these drugs and of HT. Stratified analyses will allow these factors to be taken into account to some extent.

Plans for communicating and disseminating results

We aim to publish the results of this study in a peer-reviewed journal at the earliest opportunity. Study results will also be submitted for presentation at an appropriate scientific meeting. There are no restrictions on the extent or timing of publication.

**References**

1. Rossouw JE, Anderson GL, Prentice RL, LaCroix AZ, Kooperberg C, Stefanick ML, et al. Risks and benefits of estrogen plus progestin in healthy postmenopausal women: Principal results from the women's health initiative randomized controlled trial. Journal of the American Medical Association. 2002;288(3):321-33.

2. Million Women Study Collaborators. Breast cancer and hormone-replacement therapy in the Million Women Study. The Lancet. 2003;362(9382):419-27.

3. Anderson GL, Limacher M. Effects of Conjugated Equine Estrogen in Postmenopausal Women with Hysterectomy: The Women's Health Initiative Randomized Controlled Trial. Journal of the American Medical Association. 2004;291(14):1701-12.

4. Collaborative Group on Hormonal Factors in Breast Cancer. Breast cancer and hormonal contraceptives: Collaborative reanalysis of individual data on 53 297 women with breast cancer and 100 239 women without breast cancer from 54 epidemiological studies. Lancet. 1996;347(9017):1713-27.

5. Calle EE, Heath Jr CW, Coates RJ, Liff JM, Franceschi S, Talamini R, et al. Breast cancer and hormone replacement therapy: Collaborative reanalysis of data from 51 epidemiological studies of 52,705 women with breast cancer and 108,411 women without breast cancer. Lancet. 1997;350(9084):1047-59.

6. Beral V, Bull D, Doll R, Peto R, Reeves G. Breast cancer and breastfeeding: Collaborative reanalysis of individual data from 47 epidemiological studies in 30 countries, including 50 302 women with breast cancer and 96 973 women without the disease. Lancet. 2002;360(9328):187-95.

7. Hunter DJ, Colditz GA, Hankinson SE, Malspeis S, Spiegelman D, Chen W, et al. Oral contraceptive use and breast cancer: A prospective study of young women. Cancer Epidemiology Biomarkers and Prevention. 2010;19(10):2496-502.

8. Medicines and Healthcare products Regulatory Agency (MHRA). Hormone replacement therapy: updated advice. Drug safety Update, September 2007. Last accessed 14.7.2020.

9. Prentice RL, Manson JE, Langer RD, Anderson GL, Pettinger M, Jackson RD, et al. Benefits and risks of postmenopausal hormone therapy when it is initiated soon after menopause. American Journal of Epidemiology. 2009;170(1):12-23.

10. Lyytinen HK, Dyba T, Ylikorkala O, Pukkala EI. A case-control study on hormone therapy as a risk factor for breast cancer in Finland: Intrauterine system carries a risk as well. International Journal of Cancer. 2010;126(2):483-9.

11. Fournier A. Should transdermal rather than oral estrogens be used in menopausal hormone therapy?: A review. Menopause International. 2010;16(1):23-32.

12. Tannen RL, Weiner MG, Xie D, Barnhart K. Estrogen affects post-menopausal women differently than estrogen plus progestin replacement therapy. Human Reproduction. 2007;22(6):1769-77.

13. Tannen RL, Weiner MG, Xie D, Barnhart K. A simulation using data from a primary care practice database closely replicated the women's health initiative trial. J Clin Epidemiol. 2007;60(7):686-95.

14. Opatrny L, Dell'Aniello S, Assouline S, Suissa S. Hormone replacement therapy use and variations in the risk of breast cancer. BJOG: An International Journal of Obstetrics and Gynaecology. 2008;115(2):169-75.

15. Schneider C, Jick SS, Meier CR. Risk of gynecological cancers in users of estradiol/dydrogesterone or other HRT preparations. Climacteric. 2009;12(6):514-24.

16. Bernier MO, Mikaeloff Y, Hudson M, Suissa S. Combined oral contraceptive use and the risk of systemic lupus erythematosus. Arthritis Care and Research. 2009;61(4):476-81.

AMENDMENT TO PROTOCOL 10_152

Date: 22 September 2017

Overview of amendment

The proposed amendment to protocol 10_152 is to extend and update the GPRD dataset initially approved and extracted in 2011 for the purpose of studying risk of breast cancer in users of exogenous female hormones. The original GPRD dataset provided 33,000 breast cancer cases diagnosed between 1995 and 2010, with 66,000 controls matched on year of birth (+/- 2 years), sex, general practice and observation period. Analyses related to that application have not been finalised or published to date.

Given that some years have passed since the original application, the authors wish to apply to extend the dataset to include all additional breast cancer cases and matched controls (that meet inclusion/exclusion criteria as outlined in the original protocol) for 2011 to 2017. This will allow statistical power to be maximised, and may facilitate further analyses into newer formulations of exogenous hormones, including the Mirena intra-uterine device. A full dataset refresh is therefore requested for all incident breast cancer cases meeting study eligibility criteria for years 1995 to 2017 and matched controls in a 2:1 ratio.

**Revised definitions of cases and controls**

In keeping with the above protocol, the revised definitions for cases and controls are outlined as follows. All women are required to have a minimum of 12 months up-to-standard data in the CPRD prior to index date (date of diagnosis for the case) to be eligible for study inclusion.

Case definition

Cases are defined as all female participants of the CPRD with incident diagnosis of invasive or in situ breast cancer documented between 1^st^ January 1995 and 2017 (to most recent data available), and aged 20- 79 years of age at index diagnosis. Women are excluded if they have a past history of invasive or in situ breast cancer.

The list of Read codes used to flag women with incident breast cancer diagnosis as per previous protocol is shown Appendix 1.

Control definition

Controls are defined as all women without a medical diagnosis of breast cancer documented (up to last date of data extraction for 2017). This includes any diagnoses for breast cancer prior to 1^st^ Jan 1995 and during the study period from 1^st^ Jan 1995 to 2017. Cases are therefore not eligible to act as controls in this study.

Controls will be matched in a 2:1 ratio to cases based on:

- Year of birth(+/- 2 years)
- Same general practice
- Similar period of observation as the case.

The period of observation in CPRD for controls must include the full observation time (regstart to index diagnosis) for cases, so that observation periods may be matched exactly for the purpose of analyses.

**Additional study investigators**

The chief investigator for the study is unchanged from previous, Professor Jane Green.

Additional study collaborators are outlined below:

Professor Dame Valerie Beral

Director of Epidemiology and Co-Director, Cancer Epidemiology Unit

University of Oxford

E: [pa.valerie.beral@ceu.ox.ac.uk](mailto:pa.valerie.beral@ceu.ox.ac.uk)

CV has been previously submitted to ISAC (CV number 178_15CEL)

Professor Gillian Reeves

Nuffield Department of Population Health

University of Oxford

E: [gill.reeves@ceu.ox.ac.uk](mailto:gill.reeves@ceu.ox.ac.uk)

Dr Danielle Fitzpatrick

Nuffield Department of Population Health

University of Oxford

E: [danielle.fitzpatrick@magd.ox.ac.uk](mailto:danielle.fitzpatrick@magd.ox.ac.uk)

Ms Kirstin Pirie

Nuffield Department of Population Health

University of Oxford

E: [kirstin.pirie@ceu.ox.ac.uk](mailto:kirstin.pirie@ceu.ox.ac.uk)

Mr Keith Shaw

Nuffield Department of Population Health

University of Oxford

E: [keith.shaw@ceu.ox.ac.uk](mailto:kirstin.pirie@ceu.ox.ac.uk)

**Appendix 1: List of read codes for identifying breast cancer diagnosis in CPRD**

| **Med Code** | **Read Code** | **Read Term** | **ICD-Code** |
| --- | --- | --- | --- |
| 3968 | B34..00 | Malignant neoplasm of female breast | C50 |
| 348 | B34..11 | Ca female breast |  |
| 26853 | B340.00 | Malignant neoplasm of nipple and areola of female breast | C50 |
| 23380 | B340000 | Malignant neoplasm of nipple of female breast | C50 |
| 64686 | B340100 | Malignant neoplasm of areola of female breast | C50 |
| 59831 | B340z00 | Malignant neoplasm of nipple or areola of female breast NOS | C50 |
| 31546 | B341.00 | Malignant neoplasm of central part of female breast | C50 |
| 29826 | B342.00 | Malignant neoplasm of upper-inner quadrant of female breast | C50 |
| 45222 | B343.00 | Malignant neoplasm of lower-inner quadrant of female breast | C50 |
| 23399 | B344.00 | Malignant neoplasm of upper-outer quadrant of female breast | C50 |
| 42070 | B345.00 | Malignant neoplasm of lower-outer quadrant of female breast | C50 |
| 20685 | B346.00 | Malignant neoplasm of axillary tail of female breast | C50 |
| 49148 | B347.00 | Malignant neoplasm of overlapping lesion of breast | C50 |
| 56715 | B34y.00 | Malignant neoplasm of other site of female breast | C50 |
| 95057 | B34y000 | Malignant neoplasm of ectopic site of female breast | C50 |
| 38475 | B34yz00 | Malignant neoplasm of other site of female breast NOS | C50 |
| 9470 | B34z.00 | Malignant neoplasm of female breast NOS | C50 |
| 7833 | B830.00 | Carcinoma in situ of breast | D05 |
| 10387 | B830000 | Lobular carcinoma in situ of breast | D05 |
| 18694 | B830100 | Intraductal carcinoma in situ of breast | D05 |
| 18029 | BB9..00 | [M]Ductal, lobular and medullary neoplasms |  |
| 27728 | BB90.00 | [M]Intraductal carcinoma, noninfiltrating NOS |  |
| 8351 | BB91.00 | [M]Infiltrating duct carcinoma |  |
| 21833 | BB91.11 | [M]Duct carcinoma NOS |  |
| 30189 | BB91000 | [M]Intraductal papillary adenocarcinoma with invasion |  |
| 39760 | BB91100 | [M]Infiltrating duct and lobular carcinoma |  |
| 62871 | BB92.00 | [M]Comedocarcinoma, noninfiltrating |  |
| 58131 | BB93.00 | [M]Comedocarcinoma NOS |  |
| 40359 | BB94.00 | [M]Juvenile breast carcinoma |  |
| 67701 | BB94.11 | [M]Secretory breast carcinoma |  |
| 16677 | BB9B.00 | [M]Medullary carcinoma NOS |  |
| 47920 | BB9B.11 | [M]C cell carcinoma |  |
| 50946 | BB9C.00 | [M]Medullary carcinoma with amyloid stroma |  |
| 98883 | BB9D.00 | [M]Medullary carcinoma with lymphoid stroma |  |
| 21861 | BB9E.00 | [M]Lobular carcinoma in situ |  |
| 9956 | BB9E000 | [M]Intraductal carcinoma and lobular carcinoma in situ |  |
| 12427 | BB9F.00 | [M]Lobular carcinoma NOS |  |
| 7319 | BB9G.00 | [M]Infiltrating ductular carcinoma |  |
| 32472 | BB9H.00 | [M]Inflammatory carcinoma |  |
| 12300 | BB9J.00 | [M]Paget’s disease, mammary |  |
| 60803 | BB9J.11 | [M]Paget’s disease, breast |  |
| 42542 | BB9K.00 | [M]Paget’s disease and infiltrating breast duct carcinoma |  |
| 12480 | BB9K000 | [M]Paget’s disease and intraductal carcinoma of breast |  |
| 3969 | BB9M.00 | [M]Intracystic carcinoma NOS |  |
| 60683 | BB9z.00 | [M]Ductal, lobular or medullary neoplasm NOS |  |
| 12499 | Byu6.00 | [X]Malignant neoplasm of breast |  |
| 53803 | ByuFG00 | [X]Other carcinoma in situ of breast | D05 |
| 16639 | ZV10300 | [V]Personal history of malignant neoplasm of breast |  |
