# Supplementary material for: Combined and progestagen-only hormonal contraceptives and breast cancer risk: A UK nested case–control study and meta-analysis
Source: PLoS Med. 2023 Mar 21;20(3):e1004188. doi: 10.1371/journal.pmed.1004188 (PMC10030023; doi:10.1371/journal.pmed.1004188)
Supplement: S1 File — (DOCX) [file pmed.1004188.s004.docx]

**Additional information about the Clinical Practice Research Datalink (CPRD)**

The CPRD is a computerised UK primary care database containing anonymised, linked and prospective medical records for approximately 11 million patients registered with a National Health Service (NHS) general practitioner (GP) [1]. As of 2015, approximately 7 percent of the UK population were actively participating in the CPRD [1]. Details of primary care consultations including diagnoses, referrals and prescriptions are recorded by practices and uploaded to CPRD on a monthly basis [1]. Prescriptions are coded automatically as they are generated by the GP according to their product name and British National Formulary code [2], thus providing near complete prescription records. Diagnoses are coded to the Read clinical classification system [3]. Individuals enter CPRD on the date that they register with a participating general practice, and exit when they move to a non-CPRD collaborating general practice, leave the NHS, withdraw from CPRD or die [1]. Patient and practice level measures of data quality are used to exclude patients that do not meet basic quality criteria and to determine the date at which each practice is deemed to be up to standard.

Patients within the CPRD database have been shown to be broadly representative in terms of age and sex of the wider UK population [1], and had a median duration of follow-up of 5.1 [IQR 1.8-11.1] years in 2013 [1]. The validity of medical diagnoses within the CPRD has been estimated at above 90 percent for malignancy overall [4-6], as well as for breast cancer diagnosis alone [4-7].

**List of CPRD Read codes for invasive breast cancer**

| **Read Code** | **Description** |
| --- | --- |
| B34..11 | Ca female breast |
| B34..00 | Malignant neoplasm of female breast |
| BB9G.00 | [M]Infiltrating ductular carcinoma |
| BB91.00 | [M]Infiltrating duct carcinoma |
| B34z.00 | Malignant neoplasm of female breast NOS |
| BB9J.00 | [M]Paget's disease, mammary |
| BB9F.00 | [M]Lobular carcinoma NOS |
| BB9K000 | [M]Paget's disease and intraductal carcinoma of breast |
| Byu6.00 | [X]Malignant neoplasm of breast |
| BB9B.00 | [M]Medullary carcinoma NOS |
| BB9..00 | [M]Ductal, lobular and medullary neoplasms |
| B346.00 | Malignant neoplasm of axillary tail of female breast |
| BB91.11 | [M]Duct carcinoma NOS |
| B340000 | Malignant neoplasm of nipple of female breast |
| B344.00 | Malignant neoplasm of upper-outer quadrant of female breast |
| B340.00 | Malignant neoplasm of nipple and areola of female breast |
| B342.00 | Malignant neoplasm of upper-inner quadrant of female breast |
| BB91000 | [M]Intraductal papillary adenocarcinoma with invasion |
| B341.00 | Malignant neoplasm of central part of female breast |
| BB9H.00 | [M]Inflammatory carcinoma |
| B34yz00 | Malignant neoplasm of other site of female breast NOS |
| BB91100 | [M]Infiltrating duct and lobular carcinoma |
| BB94.00 | [M]Juvenile breast carcinoma |
| B933.00 | Neoplasm of uncertain behaviour of breast |
| B345.00 | Malignant neoplasm of lower-outer quadrant of female breast |
| BB9K.00 | [M]Paget's disease and infiltrating breast duct carcinoma |
| B343.00 | Malignant neoplasm of lower-inner quadrant of female breast |
| BA03.00 | Neoplasm of unspecified nature of breast |
| BB9B.11 | [M]C cell carcinoma |
| B347.00 | Malignant neoplasm, overlapping lesion of breast |
| B34y.00 | Malignant neoplasm of other site of female breast |
| B340z00 | Malignant neoplasm of nipple or areola of female breast NOS |
| BB9z.00 | [M]Ductal, lobular or medullary neoplasm NOS |
| B340100 | Malignant neoplasm of areola of female breast |
| BB94.11 | [M]Secretory breast carcinoma |

**References**

1. Herrett E, Gallagher AM, Bhaskaran K, et al. Data Resource Profile: Clinical Practice Research Datalink (CPRD). *International journal of epidemiology BMJ*2015; 44(3): 827-36.
2. Joint Formulary Committee. British National Formulary 74th edition ed. London: BMJ Group and Pharmaceutical Press; 2017.
3. Chisholm J. The Read clinical classification. *BMJ* 1990; 300(6732): 1092.
4. Boggon R, van Staa TP, Chapman M, Gallagher AM, Hammad TA, Richards MA. Cancer recording and mortality in the General Practice Research Database and linked cancer registries. *Pharmacoepidemiology and drug safety*. 2013;22(2):168-75.
5. Herrett E, Thomas SL, Schoonen WM, Smeeth L, Hall AJ. Validation and validity of diagnoses in the General Practice Research Database: a systematic review. *British journal of clinical pharmacology* 2010; 69(1): 4-14.
6. Dregan A, Moller H, Murray-Thomas T, Gulliford MC. Validity of cancer diagnosis in a primary care database compared with linked cancer registrations in England. Population-based cohort study. *Cancer epidemiology* 2012; 36(5): 425-9.
7. Arhi CS, Bottle A, Burns EM, et al. Comparison of cancer diagnosis recording between the Clinical Practice Research Datalink, Cancer Registry and Hospital Episodes Statistics. *Cancer epidemiology* 2018; 57: 148-57.
