# Supplementary material for: Combined and progestagen-only hormonal contraceptives and breast cancer risk: A UK nested case–control study and meta-analysis
Source: PLoS Med. 2023 Mar 21;20(3):e1004188. doi: 10.1371/journal.pmed.1004188 (PMC10030023; doi:10.1371/journal.pmed.1004188)
Supplement: S2 File — (DOCX) [file pmed.1004188.s005.docx]

**Risk of breast cancer in never users of hormonal contraceptives in Western countries**

We used age-specific breast cancer registration rates in England in 2019 [1] to estimate breast cancer risk, by age, expressed as incidence rates of invasive breast cancer per 100,000 women over a 5 year period. The rates in England are typical of rates in Northern Europe, Western Europe, North America, Australia, and New Zealand [2].

Age-specific estimates of breast cancer risk in never-users of hormonal contraceptives were then calculated based on the overall age-specific risks, age-specific prevalences of current or recent use of hormonal contraceptive use, and the estimated odds ratios for breast cancer associated with current or recent use of hormonal contraceptives (Figure 1).

The risks below would apply to pre-menopausal women aged younger than 50 years.

**Estimated breast cancer risk for women of average weight in Western countries in 2019**

| **Age** | **5-year breast cancer risk per 100,000 women** | **Estimated 5-year breast cancer risk per 100,000 never users of hormonal contraceptives** |
| --- | --- | --- |
| 16-20 years* | 2.6 | 2.2 |
| 20-24 years | 9.0 | 7.7 |
| 25-29 years | 56.5 | 48.4 |
| 30-34 years | 160.0 | 142.9 |
| 35-39 years | 351.0 | 313.4 |
| 40-44 years | 654.5 | 616.0 |
| 45-49 years | 1087.5 | 1023.5 |

* 5-year risk for women aged 16-20 years = (0.8 x 5-year risk for women aged 16-19 years) + (0.2 x 5-year risk for women aged 20-24 years)

**References**

1. NHS Digital. Cancer registration statistics, England 2019. https://digital.nhs.uk/data-and-information/publications/statistical/cancer-registration-statistics/england-2019 (Accessed: August 10, 2022).
2. WHO International Agency for Research on Cancer. C15 Plus. Cancer incidence in five continents time trends. Graphs: time trends by age. ci5.iarc.fr/CI5plus/Pages/graph2_sel.aspx (accessed Nov 22 2019)
