# Supplementary material for: Combined and progestagen-only hormonal contraceptives and breast cancer risk: A UK nested case–control study and meta-analysis
Source: PLoS Med. 2023 Mar 21;20(3):e1004188. doi: 10.1371/journal.pmed.1004188 (PMC10030023; doi:10.1371/journal.pmed.1004188)
Supplement: S1 Table — (DOCX) [file pmed.1004188.s006.docx]

**S1 Table: Sensitivity analyses of odds ratios for breast cancer associated with any use of hormonal contraceptives compared to women with no prescriptions for hormonal contraceptives.**

OR = Odds ratio; CI = Confidence interval. P-values are from Wald tests.

|  | **Cases exposed/**  **not exposed** | **OR (95% CI)** | **P-value** |
| --- | --- | --- | --- |
| Sensitivity analysis: |  |  |  |
| Defining exposure as 2+ hormonal contraceptive prescriptions | 3461 / 5303 | 1.26 (1.18-1.34) | <0.001 |
| Observation window ≥ 5 years | 3175 / 2658 | 1.25 (1.16-1.34) | <0.001 |
| Excluding women with hysterectomy, bilateral oophorectomy or tubal ligation | 3989 / 4718 | 1.27 (1.19-1.35) | <0.001 |
| Complete data on adjustment variables | 2923 / 3422 | 1.22 (1.12-1.32) | <0.001 |
