# Supplementary material for: Combined and progestagen-only hormonal contraceptives and breast cancer risk: A UK nested case–control study and meta-analysis
Source: PLoS Med. 2023 Mar 21;20(3):e1004188. doi: 10.1371/journal.pmed.1004188 (PMC10030023; doi:10.1371/journal.pmed.1004188)
Supplement: S2 Table — (DOCX) [file pmed.1004188.s007.docx]

**S2 Table: Odds ratios for breast cancer associated with one or more prescriptions for different British National Formulation (BNF) [1] medication types during the observation window**.

OR = Odds ratio; CI = Confidence interval. P-values are from Wald tests.

|  | **Cases exposed** | **OR (95% CI)** | **p-value** |
| --- | --- | --- | --- |
| Any hormonal contraceptive | 4195 | 1.25 (1.18-1.33) | p<0.001 |
| Non-sedating anti-histamines (BNF 3.4.1.1) | 1185 | 1.01 (0.95-1.08) | p=0.7 |
| Antibacterials, in eye preparation (BNF 11.3.1) | 1554 | 1.04 (0.97-1.11) | p=0.3 |
| Corticosteroids, for respiratory conditions (BNF 3.2) | 962 | 1.02 (0.94-1.11) | p=0.6 |

**References**

1. Joint Formulary Committee. British National Formulary 74th edition ed. London: BMJ Group and Pharmaceutical Press; 2017.
