# Supplementary material for: Combined and progestagen-only hormonal contraceptives and breast cancer risk: A UK nested case–control study and meta-analysis
Source: PLoS Med. 2023 Mar 21;20(3):e1004188. doi: 10.1371/journal.pmed.1004188 (PMC10030023; doi:10.1371/journal.pmed.1004188)
Supplement: S3 Table — (DOCX) [file pmed.1004188.s008.docx]

**S3 Table. Summary of studies meeting inclusion/exclusion criteria for meta-analyses of breast cancer risk associated with current and/or recent use of progestagen-only contraceptives**

| **Publication author, year** | **Study name, design, and country**  **(years of case ascertainment)** | **Age at breast cancer diagnosis (years)** | **Exposure ascertainment and definition** | **Adjustment or stratification variables** | **Time since last use** | **Number of breast cancer cases with recent/never use and RR (95%CI)** |
| --- | --- | --- | --- | --- | --- | --- |
| Collaborative Group on Hormonal Factors in Breast Cancer (CGHFBC), 1996 [1] | Meta-analysis of studies published prior to 1996; most studies of progestagen-only use were retrospective | 15-90; median 49 | Progestagen-only oral [injectable] use vs never use of progestagen-only oral [injectable] | Study, age at diagnosis, parity, age at first birth, age at which risk of conception ceased | <5 years  (<7 years for cohort studies) | **Progestagen pill:**  375/29625; RR=1.17 (95% CI 0.99-1.38)  **Injectable:**  137/25612; RR=1.17 (95% CI 0.92-1.49) |
| Shapiro et al, 2000 [2] | Retrospective case-control study, hospital controls, South Africa  (1994-1997) | 20-54; median 44 | Nurse-administered questionnaire: current/recent use of progestagen-only injection vs never use of progestagen-only injection | Age, ethnic group, socioeconomic status, use of combined oral contraceptives. Authors report nil change in RR estimates on adjusting for family history of breast cancer, parity, age at first birth, breast feeding, benign breast disease, and region | <5 years | **Injectable:**  **Current:** 65/166; RR=1.6 (95% CI 1.1-2.3)  **Last use 1-4 years prior:** 59/166; RR=1.0 (95% CI 0.7-1.5)  **Use in the last 5 years^d^:** 124/166; RR=1.28 (95% CI 0.95-1.71) |
| Kumle et al, 2002 [3]^a^ | Women’s Lifestyle and Health Study, prospective cohort, Norway and Sweden  (1991-1999) | 30-57; median 47 | Self-administered questionnaire at baseline: exclusive use of progestagen-only pill vs never-use of combined oral contraceptives or progestagen-only pill | Age at enrolment, region, age at menarche, age at first birth, parity, number of full-term pregnancies, use of HRT, family history of breast cancer, duration of breastfeeding, BMI, menopausal status | Current or recent use (<1 year prior) at entry; maximum 8 years follow-up | **Progestagen pill:**  25/261; RR=1.6 (95% CI 1.0-2.4) |
| Strom et al, 2004 [4]^b^ | Women’s CARE Study, US retrospective case-control study, population controls  (1994-1998) | 35-64, mean 50 (results reported unchanged in a subgroup of pre-menopausal women only) | In-person interviews: progestagen-only injectable [implant] use vs never-use of progestagen-only injectable [implant] | Age, race, study site. Authors report nil change in RR estimates, but increased variability, on adjusting for reproductive factors including menopausal status, age at menarche or menopause, parity, BMI, family history etc. | Current use (≤1 year ago) reported for injectables; Ever-use only reported for implant | **Injectable (current use):**  15/4480; RR=0.67 (95% CI 0.35-1.30)  **Implant (ever use):**  5/4569; RR=0.67 (95% CI 0.21-2.13) |
| Sweeney et al, 2007 [5] | 4-Corners Study, Retrospective case-control study with population controls, US  (1999-2004) | Median age 54 (cases and controls) | In-person interviews: recent progestagen-only injection [implant] vs never progestagen-only injection [implant] | Age, study centre, education, family history of breast cancer, alcohol, language acculturation, number of births, years since last birth, age at menopause, use of oral contraceptives, recent use of hormone replacement therapy, ethnicity | **Injectable:** <5 years  **Implant:** <10 years since first use (for 87% of users) | **Injectable:**  29/2224; RR=1.14 (95% CI 0.66-1.95)  **Implant:**  15/2303; RR=8.59 (95% CI 1.92-38.4) |
| Dinger et al, 2011 [6] | Retrospective population-based case-control study, Finland/Germany  (2000-2007) | <50; mean 44.5 | Self-administered questionnaires: current progestagen-releasing IUD vs current copper IUD | BMI, family history of breast cancer, parity, age at first birth, breastfeeding, age at menarche, HRT use, oral contraceptive use, physical activity, educational level, smoking, alcohol, duration of IUD use, long-term use of concomitant medication | Current use | **IUD:**  RR=0.88 (95% CI 0.49-1.59) |
| Li et al, 2012 [7] | Retrospective population-based case control study, US  (2004-2010) | 20-44 | In-person interviews: recent progestagen-only injection (one or more shot within 5 years of reference date) vs never progestagen-only injection | Age, reference year, family history of breast cancer, BMI, parity, duration of oral contraceptive use, screening mammography (no significant difference on adjusting for education, income, race, age at first live birth) | <5 years | **Injectable:**  36/907; RR=1.50 (95% CI 0.90-2.70) |
| Urban et al, 2012 [8] | Johannesburg Cancer Case Control Study, retrospective, hospital controls  (1995-2006) | 18-79; median 44 in cases who used a progestagen-only injectable | Nurse-administered interviews: ever progestagen-only injection in the last 10 years (and never used oral contraceptives) vs never progestagen-only injection or oral contraceptives | Age, year, education, smoking, alcohol consumption, parity/age at first birth, number of sexual partners, urban/rural residence, province of birth | <10 years | **Injectable:**  186/856; RR=1.83 (95% CI 1.31-2.55) |
| Morch et al, 2017 [9]^c^ | Danish Sex Hormone Register Study, nationwide prospective cohort study in Denmark (1995 – 2012) | 15-49 | Prescription records: current or recent use (discontinued in the previous 6 months) of each progestagen-only type vs never-use of any hormonal contraceptive | Age, calendar year, level of education, parity, PCOS, endometriosis, family history of premenopausal breast or ovarian cancer | < 6 months  (recent use defined as hormonal contraceptive discontinued within the previous 6 months) | **Progestagen pill:**  **Norethisterone**: 78/5955; RR=1.00 (95% CI 0.80-1.25)  **Levonorgestrel**: 16/5955; RR=1.93 (95% CI 1.18-3.16)  **Desogestrel**: 42/5955; RR=1.18 (95% CI 0.87-1.60)  **Any progestagen pill^d^**:  136/5955; RR=1.14 (95% CI 0.96-1.35)  **Implant:**  9/5955; RR=0.93 (95% CI 0.48-1.79)  **Injectable:**  5/5955; RR=0.95 (95% CI 0.40-2.29)  **IUD:**  571/5955; RR=1.21 (95% CI 1.11-1.33) |
| Jareid et al, 2018 [10] | Norwegian Women and Cancer Study (NOWAC), prospective population-based cohort (1991-2015) | Median 52 at entry (mean 12.5 years follow-up) | Self-administered questionnaires: current progestagen-only IUD vs never progestagen-only IUD | Age at start of follow-up, BMI, physical activity, family history of breast cancer, oral contraceptive use, menopausal status at start of follow-up | Current use | **IUD:**  RR=0.97 (95% CI 0.80-1.19) |
| Busund et al, 2018 [11] | Norwegian Women and Cancer Study (NOWAC)- prospective population-based cohort (1991-2015) | 30-53; mean 41 at baseline (mean 7.8 years follow-up) | Self-administered questionnaires: current progestagen-only pill vs never oral contraceptive use | BMI, history of breast cancer in mother, age at menarche, alcohol consumption, parity and age at first birth, combined oral contraceptive use | Current use | **Progestagen pill:**  28/379; RR=1.42 (95% CI 0.90-2.26) |
| Hultstrand et al, 2022 [12] | Prospective nation-wide cohort study, Sweden (2005-2017) | 15-45 | Redeemed prescription records (Swedish Prescribed Drug Register): current or recent use of each progestagen-only type vs never-use of any hormonal contraceptive | Age, level of education, place of birth, age at first birth, number of children, prior ovulation simulating treatment, infertility, PCOS, endometriosis. Additional adjustment for BMI and smoking was done in a subset of parous women only (37% of cohort). | Current use | **Progestagen pill**:  398/1419; RR=1.40 (95% CI 1.26-1.56)  **Implant:**  78/1419; RR=1.31 (95% CI 1.05-1.64)  **Injectable:**  50/1419; RR=0.74 (95% CI 0.56-0.97)  **IUD:**  543/1419; RR=1.21 (95% CI 1.01-1.33) |

BMI = Body mass index; HRT = Hormone replacement therapy; IUD = Intra-uterine device; PCOS = Polycystic ovarian syndrome; RR = Relative risk; CI = Confidence interval

^a^ Possible overlap of Norwegian data 1991-1999 with Busund et al. (NOWAC)

^b^ Injectable users: 127/204 depo provera, 77/204 unknown type

^c^ Progestagen-releasing IUD assumed to be used for 4 years (unless known pregnancy or other contraceptive prescribed sooner)

^d^ Derived by combining published RRs

**References**

1. Collaborative Group on Hormonal Factors in Breast Cancer. Breast cancer and hormonal contraceptives: Collaborative reanalysis of individual data on 53 297 women with breast cancer and 100 239 women without breast cancer from 54 epidemiological studies. *Lancet*. 1996;347(9017):1713-27.

2. Shapiro S, Rosenberg L, Hoffman M, Truter H, Cooper D, Rao S, et al. Risk of breast cancer in relation to the use of injectable progestogen contraceptives and combined estrogen/progestogen contraceptives. *American Journal of Epidemiology*. 2000;151(4):396-403.

3. Kumle M, Weiderpass E, Braaten T, Persson I, Adami HO, Lund E. Use of oral contraceptives and breast cancer risk: The Norwegian-Swedish women's lifestyle and health cohort study. *Cancer Epidemiology Biomarkers and Prevention*. 2002;11(11):1375-81.

4. Strom BL, Berlin JA, Weber AL, Norman SA, Bernstein L, Burkman RT, et al. Absence of an effect of injectable and implantable progestin-only contraceptives on subsequent risk of breast cancer. *Contraception*. 2004;69(5):353-60.

5. Sweeney C, Giuliano AR, Baumgartner KB, Byers T, Herrick JS, Edwards SL, et al. Oral, injected and implanted contraceptives and breast cancer risk among U.S. Hispanic and non-Hispanic white women. *International Journal of Cancer*. 2007;121(11):2517-23.

6. Dinger J, Bardenheuer K, Minh TD. Levonorgestrel-releasing and copper intrauterine devices and the risk of breast cancer. *Contraception*. 2011;83(3):211-7.

7. Li CI, Beaber EF, Tang MT, Porter PL, Daling JR, Malone KE. Effect of depo-medroxyprogesterone acetate on breast cancer risk among women 20 to 44 years of age. *Cancer Res*. 2012;72(8):2028-35.

8. Urban M, Banks E, Egger S, Canfell K, O'Connell D, Beral V, et al. Injectable and oral contraceptive use and cancers of the breast, cervix, ovary, and endometrium in black South African women: case-control study. *PLoS Med*. 2012;9(3):e1001182.

9. Mørch LS, Skovlund CW, Hannaford PC, Iversen L, Fielding S, Lidegaard Ø. Contemporary Hormonal Contraception and the Risk of Breast Cancer. *N Engl J Med*. 2017;377(23):2228-39.

10. Jareid M, Aarflot M, Bovelstad HM, Lund E, Braaten T, Thalabard J-C. Levonorgestrel-releasing intrauterine system use is associated with a decreased risk of ovarian and endometrial cancer, without increased risk of breast cancer. Results from the NOWAC Study. *Gynecologic Oncology*. 2018;149(1):127-32.

11. Busund M, Bugge NS, Braaten T, Waaseth M, Rylander C, Lund E. Progestin-only and combined oral contraceptives and receptor-defined premenopausal breast cancer risk: The Norwegian Women and Cancer Study. *Int J Cancer*. 2018;142(11):2293-302.

12. Niemeyer Hultstrand J, Gemzell-Danielsson K, Kallner HK, Lindman H, Wikman P, Sundström-Poromaa I. Hormonal contraception and risk of breast cancer and breast cancer in situ among Swedish women 15–34 years of age: A nationwide register-based study. *The Lancet Regional Health - Europe*. 2022;21.
