# Supplementary material for: Combined and progestagen-only hormonal contraceptives and breast cancer risk: A UK nested case–control study and meta-analysis
Source: PLoS Med. 2023 Mar 21;20(3):e1004188. doi: 10.1371/journal.pmed.1004188 (PMC10030023; doi:10.1371/journal.pmed.1004188)
Supplement: S4 Table — (DOCX) [file pmed.1004188.s009.docx]

**S4 Table: Estimated excess incidence of breast cancer per 100,000 women within England associated with five years use of any oral contraceptive, by age at first use.**

|  | **5-year breast cancer risk per 100,000 non users** | **5-year breast cancer**  **risk per 100,000 users** | **Excess risk of breast cancer with 5 years use of OCs** | | |
| --- | --- | --- | --- | --- | --- |
|  |  |  | **Relative risk**  **(RR)** | **Proportional excess risk**  **(RR-1)** | **Absolute excess per 100,000 users** |
| **Use from 16 to 20** |  |  |  |  |  |
| 16-20 (current use) | 2.2 | 2.8 | 1.27 | 0.27 | 0.6 |
| 21-25 (last use 1-4 years ago)* | 15.8 | 18.3 | 1.16 | 0.16 | 2.5 |
| 26-30 (last use 5-9 years ago)* | 66.1 | 71.4 | 1.08 | 0.08 | 5.3 |
| **TOTAL** | **84.1** | **92.5** |  |  | **8.4** |
| **Excess incidence:** |  |  |  |  | **1 per 12,000 users** |
| **Use from 20 to 24** |  |  |  |  |  |
| 20-24 (current use) | 7.7 | 9.8 | 1.27 | 0.27 | 2.1 |
| 25-29 (last use 1-4 years ago) | 48.4 | 56.1 | 1.16 | 0.16 | 7.7 |
| 30-34 (last use 5-9 years ago) | 142.9 | 154.3 | 1.08 | 0.08 | 11.4 |
| **TOTAL** | **199.0** | **220.2** |  |  | **21.2** |
| **Excess incidence:** |  |  |  |  | **1 per 4500 users** |
| **Use from 25 to 29** |  |  |  |  |  |
| 25-29 (current use) | 48.4 | 61.5 | 1.27 | 0.27 | 13.1 |
| 30-34 (last use 1-4 years ago) | 142.9 | 165.8 | 1.16 | 0.16 | 22.9 |
| 35-39 (last use 5-9 years ago) | 313.4 | 338.5 | 1.08 | 0.08 | 25.1 |
| **TOTAL** | **504.7** | **565.8** |  |  | **61.1** |
| **Excess incidence:** |  |  |  |  | **1 per 1500 users** |
| **Use from 30 to 34** |  |  |  |  |  |
| 30-34 (current use) | 142.9 | 181.5 | 1.27 | 0.27 | 38.6 |
| 35-39 (last use 1-4 years ago) | 313.4 | 363.5 | 1.16 | 0.16 | 50.1 |
| 40-44 (last use 5-9 years ago) | 616.0 | 665.3 | 1.08 | 0.08 | 49.3 |
| **TOTAL** | **1072.3** | **1210.3** |  |  | **138.0** |
| **Excess incidence:** |  |  |  |  | **1 per 700 users** |
| **Use from 35 to 39** |  |  |  |  |  |
| 35-39 (current use) | 313.4 | 398.0 | 1.27 | 0.27 | 84.6 |
| 40-44 (last use 1-4 years ago) | 616.0 | 714.6 | 1.16 | 0.16 | 98.6 |
| 45-49 (last use 5-9 years ago) | 1023.5 | 1105.4 | 1.08 | 0.08 | 81.9 |
| **TOTAL** | **1952.9** | **2218.0** |  |  | **265.1** |
| **Excess incidence:** |  |  |  |  | **1 per 400 users** |

* Estimated 5-year risks per 100,000 women in age ranges 21-25, and 26-30 were calculated from appropriate weighted averages of the corresponding 5-years risks in age ranges 20-24, 25-29, and 30-34 (see S2 File)
