# Supplementary material for: Combined and progestagen-only hormonal contraceptives and breast cancer risk: A UK nested case–control study and meta-analysis
Source: PLoS Med. 2023 Mar 21;20(3):e1004188. doi: 10.1371/journal.pmed.1004188 (PMC10030023; doi:10.1371/journal.pmed.1004188)
Supplement: S1 Fig — (DOCX) [file pmed.1004188.s010.docx]

**S1 Figure: Meta-analysis of studies examining associations between current or recent use of progestagen-only contraceptives and invasive breast cancer diagnosis: PRISMA flow diagram**

Records identified through database searching
(n = 3271)

## Screening

## Included

## Eligibility

## Identification

Additional records identified through other sources
(n = 0)

Records after duplicates removed
(n = 2718)

Records screened (titles and abstracts)
(n = 2718)

Records excluded
(n = 2447)

Full-text articles assessed for eligibility
(n = 271)

**Full-text articles excluded, with reasons (n = 259)**

- Editorial, commentary, or descriptive review (n = 90)
- No risk ratios reported for contraceptive use and breast cancer (n = 36)
- No data on progestagen-only contraceptives, or where method of progestagen-only contraception was not specified (n = 93)
- Methodology (no valid control group) (n = 6)
- More recent results published in a different paper (n = 12)
- Systematic review/meta-analysis (n = 17)
- Not current/recent use (n = 3)
- Post-menopausal women only (n = 2)

Studies included in quantitative synthesis (meta-analysis)
(n = 12)
