# Supplementary material for: Combined and progestagen-only hormonal contraceptives and breast cancer risk: A UK nested case–control study and meta-analysis
Source: PLoS Med. 2023 Mar 21;20(3):e1004188. doi: 10.1371/journal.pmed.1004188 (PMC10030023; doi:10.1371/journal.pmed.1004188)

**S2 Figure: Meta-analysis of the relative risk for breast cancer associated with current or recent use of progestagen-only contraceptives in various subgroups**

CI = Confidence interval; HC = Hormonal contraceptive; IUD = Intra-uterine device.

^a^ Studies which include pre-menopausal women only. ^b^ Reference group had no prior use of progestagen-only or combined oral contraceptives.


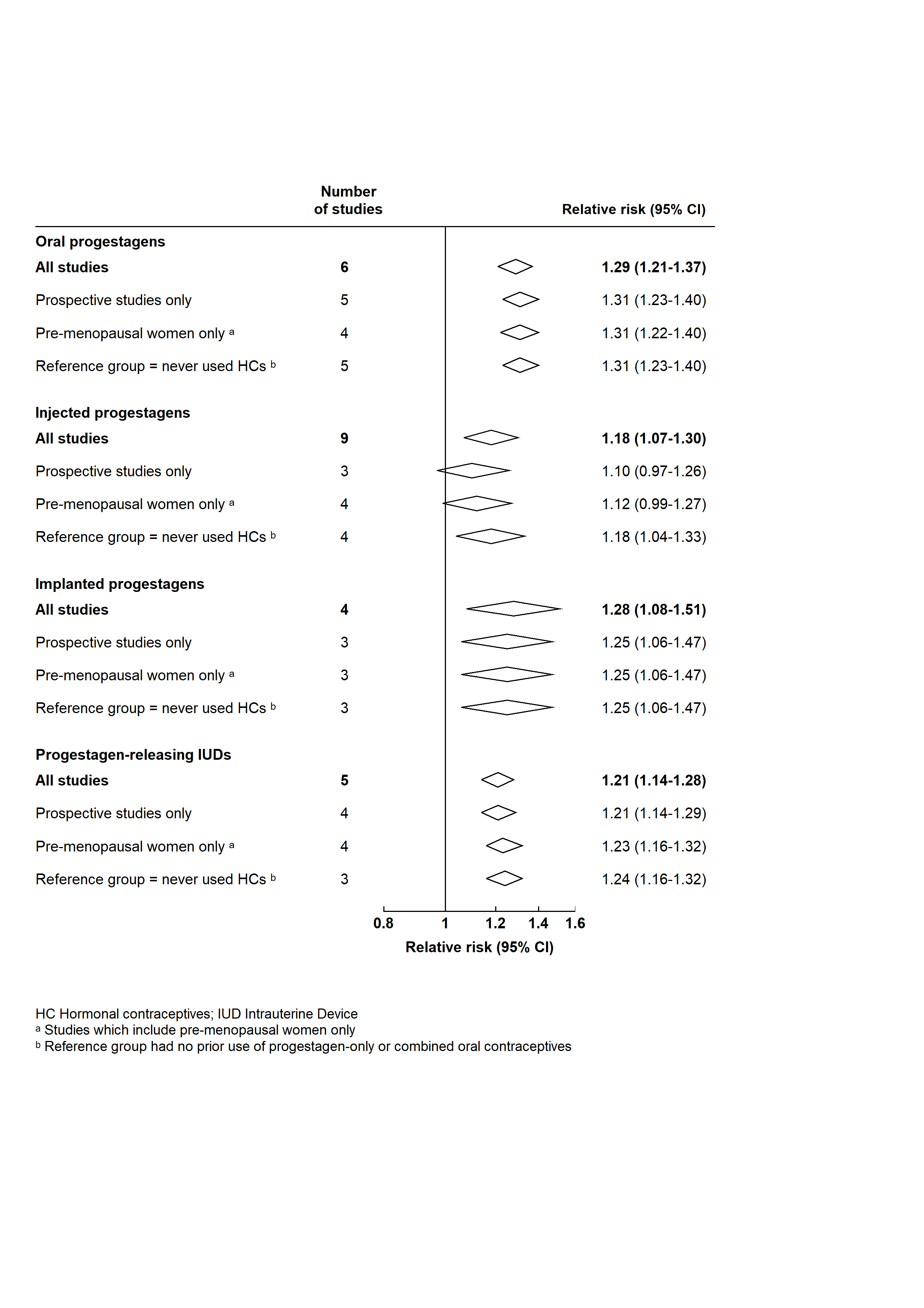

Supplement: S2 Fig — (DOCX) [file pmed.1004188.s011.docx]
