# Supplementary material for: Combined and progestagen-only hormonal contraceptives and breast cancer risk: A UK nested case–control study and meta-analysis
Source: PLoS Med. 2023 Mar 21;20(3):e1004188. doi: 10.1371/journal.pmed.1004188 (PMC10030023; doi:10.1371/journal.pmed.1004188)

**S3 Figure: Funnel plots of the log relative risk for breast cancer associated with current or recent use of four progestagen-only contraceptive types**

RR = Relative risk; SE = Standard error; IUD = Intra-uterine device.


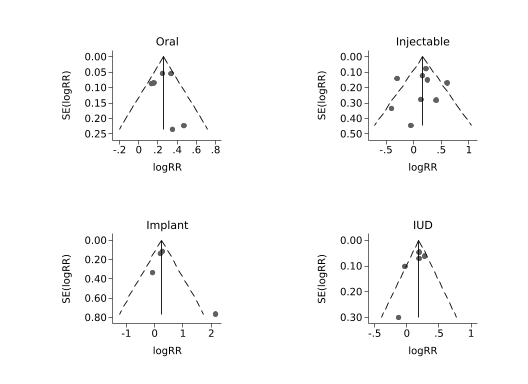

Supplement: S3 Fig — (DOCX) [file pmed.1004188.s012.docx]
